# Long term outcomes of phase I/II study of palliative triple metronomic chemotherapy in platinum-refractory/early failure oral cancer

**DOI:** 10.1016/j.lansea.2023.100143

**Published:** 2023-03-01

**Authors:** Sachin Babanrao Dhumal, Vijay Patil, Deevyashali Parekh, Vanita Noronha, Nandini Menon, Zoya Peelay, Kavita Prakash Nawale, Kumar Prabhash

**Affiliations:** a Department of Radiation Oncology, Advanced Centre for Treatment, Research and Education in Cancer (ACTREC), Homi Bhabha National Institute (HBNI) Mumbai, India; b Department of Medical Oncology, Tata Memorial Hospital, Homi Bhabha National Institute (HBNI) Mumbai, India

**Keywords:** Triple metronomic chemotherapy, Long term survival, Head neck cancer

## Abstract

**Background:**

Triple metronomic chemotherapy is one of the options of treatment in platinum-refractory/early failure oral cancer. However, long term outcomes with this regimen are unknown.

**Methods:**

Adult patients with platinum-refractory/early-failure oral cancer were enrolled in the study. Patients were administered triple metronomic chemotherapy ie erlotinib 150 mg once daily celecoxib 200 mg twice daily and methotrexate weekly (phase 1 in variable dose 15-6 mg/m^2^ & 9 mg/m^2^ in phase 2), all taken orally till progression of disease or development of intolerable adverse events. The primary objective was to estimate the long-term overall survival and factors impacting it. The Kaplan Meier method was used for time-to-event analysis. Cox proportional hazard model was used to identify factors impacting overall survival (OS) and progression-free survival (PFS). The factors included in the model were age, sex, Eastern Cooperative Oncology Group - performance status (ECOG PS), tobacco exposure and a subsite of primary and circulating endothelial cell levels at baseline. A p-value of 0.05 was considered significant. Clinical trials information: CTRI/2016/04/006834.

**Results:**

A total of 91 patients were recruited (15 in phase 1 & 76 in phase 2), the median follow-up was 41 months and 84 events of death had occurred. The median OS was 6.7 months (95% CI 5.4–7.4). The 1-year, 2-years and 3-year OS’ were 14.1% (95% CI 7.8–22.2), 5.9% (95% CI 2.2–12.2) and 5.9% (95% CI 2.2–12.2) respectively. The only factor favorably impacting OS was the detection of circulating endothelial cells at baseline (HR = 0.46; 95% CI 0.28–0.75, P = 0.0020). The median PFS was 4.3 months (95% CI 4.1–5.1) and the 1-year PFS was 13.0% (95% CI 6.8–21.2). The factors with statistically significant impact on PFS were detection of circulating endothelial cells at baseline (HR = 0.48; 95% CI 0.30–0.78, P = 0.0020) and no tobacco exposure at baseline (HR = 0.51; 95% CI 0.27–0.94, P = 0.030).

**Interpretation:**

The long-term outcomes with triple oral metronomic chemotherapy ie erlotinib, methotrexate and celecoxib are unsatisfactory. Detection of circulating endothelial cells at baseline is a biomarker predicting efficacy of this therapy.

**Funding:**

The study was funded by an intramural grant from Tata Memorial Center Research Administration Council (TRAC) and Terry Fox foundation.

**Research in context**

**Evidence before this study**

We searched the PubMed database for keywords ‘platinum refractory’, ‘oral cancer’ and ‘long term’ and only found 1 paper by Ferris et al. titled “Nivolumab vs investigator's choice in recurrent or metastatic squamous cell carcinoma of the head and neck: 2-year long-term survival update of CheckMate 141 with analyses by tumor PD-L1 expression.” Published in Oral Oncology in 2018. Given that Nivolumab and Pembrolizumab have robust data in treatment of platinum refractory oral cancer and long-term survival data only exists for Nivolumab, we decided to do this study for triple metronomic chemotherapy. Triple metronomic chemotherapy is one of the options of treatment in platinum-refractory/early failure oral cancer. However, long term outcomes with this regimen are currently lacking evidence.

**Added value of this study**

A total of 91 patients were recruited. The median OS was 6.7 months (95% CI 5.4–7.4).The 1-year, 2-years and 3-year OS’ were 14.1% (95% CI 7.8–22.2), 5.9% (95% CI 2.2–12.2) and 5.9% (95% CI 2.2–12.2) respectively. The median PFS was 4.3 months (95% CI 4.1–5.1) and the 1-year PFS was 13.0% (95% CI 6.8–21.2). . The only factor favorably impacting OS was the detection of circulating endothelial cells at baseline. The median PFS was 4.3 months. The factors with statistically significant impact on PFS were detection of circulating endothelial cells at baseline and no tobacco exposure at baseline. Per the aforementioned study by Ferris et all, Nivolumab had a 24-month OS rate of 16.9% and demonstrated OS benefit across patients with tumor PD-L1 expression ≥1% (HR [95% CI] = 0.55 [0.39–0.78]) and <1% (HR [95% CI] = 0.73 [0.49–1.09]), and regardless of tumor HPV status. The 24-month OS rate of 16.9% was similar to the 14.1% from our study.

**Implications of all the available evidence**

Currently reported systemic treatment options in platinum refractory oral cancer are Nivolumab and Pembrolizumab. However, due to the hurdles in access to these options in low and middle-income countries, we opted to study long-term survival data for triple metronomic chemotherapy in these patients. This study was from a single-center and hence can't provide a definitive conclusion however it shows us real-world data on an accessible therapy in platinum-refractory/early failure oral cancer in an LMIC. The factors with statistically significant impact on PFS were detection of circulating endothelial cells at baseline and no tobacco exposure at baseline. These results as well as other such studies performed from around the world will give us robust survival data from this clinical scenario and hence advise decisions regarding its utility in the future.

## Introduction

Oral cavity cancers presenting at an advanced stage are a major burden in India.^1^ Surgical resection of the tumor followed by the radiotherapy is the main treatment for oral cavity carcinomas. After surgical resection and chemoradiotherapy, nearly half of these patients develop locoregional and/or distant metastatic disease within 2 years. Treatment options for these patients include salvage surgical resection or palliative therapy.^2^ In the results of a single center Phase III oral cancer adjuvant trial (OCAT), 900 patients of squamous cell carcinoma of the oral cavity who were treated with adjuvant radiotherapy post surgery, locoregional recurrence was seen in almost 60% of patients.^3^ Immunotherapeutic agent nivolumab has shown long term benefit in the 2-year survival as compared to conventional chemotherapy in recurrent, metastatic head and neck cancer.^4^, ^5^, ^6^ Targeted therapy (eg Cetuximab) has been compared with platinum-based chemotherapy in head and neck cancer and has shown superior outcomes reports showing improved long term survival in head and neck cancer.^7^, ^8^, ^9^ The use of immunotherapy and targeted therapies in the low and middle income countries (LMICs) is limited due to their non-affordability.^10^^,^^11^ Triple metronomic chemotherapy with celecoxib 200 mg, methotrexate 9 mg/m^2^ and an oral tyrosine kinase inhibitor (Erlotinib 150 mg) had shown a response in platinum refractory or early failure oral cavity cancers but there is sparse data regarding its efficacy on long term overall survival.^12^ The present study is done to assess the long term outcome of triple metronomic chemotherapy (TMC) in advanced, platinum-refractory, early failure oral cavity cancer.

## Methods

### Study conduct and design

This was investigator-initiated, phase I-II, open label, single arm study which was approved by the institutional ethics committee of Tata Memorial Centre and was registered with the Clinical trial registry of India (CTRI/2016/04/006834). All patients provided written informed consent prior to participation in the study. The study was conducted in accordance with good clinical practice guidelines and the principles of declaration of Helsinki. The study was monitored periodically by the institute's data safety and monitoring board.

Recruitment of the study was from April 26, 2017 to October 1, 2018. Cut-off date for data analysis was 7th Feb 2022.

### Participants and treatment allocation

The study participants include histologically-confirmed squamous cell cancers of the oral cavity with at least one measurable lesion that had either progressed within 6 months of prior platinum based therapy or within 1 month of local therapy (surgery or radiation). The additional inclusion criteria were adult patients (Age ≥ 18 years), Eastern Cooperative Oncology Group performance status score of 0, 1, or 2 (on a scale from 0 to 5, with 0 indicating that the patient is fully active and higher scores indicating greater disability), life expectancy of less than 6 months and normal organ functions as per the protocol (supplementary appendix). Patients who had primary in salivary glands or were receiving any other investigational drugs or those having difficulty in swallowing or those with uncontrolled comorbidities or pregnant or breastfeeding women were excluded. Patients already receiving long term COX-2 inhibitors or methotrexate for any other health ailment were also excluded.

### Study procedure

Patients received erlotinib 150 mg (fixed dose) per oral once daily, capsule celecoxib 200 mg (fixed dose) per oral twice daily and oral weekly methotrexate. Patients on this metronomic chemotherapy regimen were monitored for response and toxicity at C1D8, D30 and subsequently monthly till progression. The response assessment scan was done on day 30 and then monthly till progression. Response assessment was done in accordance with the Response Evaluation Criteria in Solid Tumours (RECIST) version 1.1 The adverse events were recorded in accordance with the Common Terminology Criteria for Adverse Events (CTCAE) version 4.03. The criteria for dose reduction and dose delay provided in the study protocol (supplementary appendix). The chemotherapy regimen was continued either till development of intolerable side effects or progression of disease. The post progression treatment was in accordance with institutional standards and all patients were followed up till death. Circulating endothelial cells (EC) quantification was performed by flow cytometry at least 1 day before administration of oral therapy, then at day 8, day 30 and at each month till progression. The details about the procedure used are provided in the study protocol. The blood was processed within 24 h of collection preferably within 4 h. Following 6 markers were used in combination.

1. CD 146 (Endothelial cell marker, also seen on T cells)
2. CD 45 (Antigen present on T cell but not on endothelial cells)
3. CD 34 (Endothelial and stem cell marker)
4. CD 31 (Endothelial cell marker but absent in stem cell)
5. CD 133 (progenitor cell marker)
6. Viability stain (7 AAD) (apoptosis marker)

FACS Calibur was used for the flow cytometry analysis. The population of EC and Endothelial progenitor cells (EPC) were identified as; CECs: CD 146 (+), CD 45 (-/dim), CD 34 (+), CD 31 (+) and CD 133 (−) and Endothelial progenitor cells: CD 146 (+), CD 45 (-/dim), CD 34 (+), CD 31 (+) and CD 133 (+).

### Endpoints

This analysis focused on long term outcomes for patients. Hence, we opted to measure OS at 1, 2 and 3 year timepoints along with progression-free survival (PFS) at the same time points. The intention-to-treat (ITT) analysis was applicable for estimating PFS and OS. The progression-free survival (PFS) was defined as the time interval between the date of enrollment on the study to the date of progression or death, whichever was earlier. Patients who did not have any of these events were censored at last follow up. The overall survival (OS) was defined as the time interval between date of enrollment on the study to the date of death. Patients who did not have this event were censored at last follow up.

### Statistical analysis

Statistical Package for the Social Sciences IBM SPSS Statistics for Windows, version 20 (IBM Corp., Armonk, N.Y., USA) SPSS version 20 and Rstudio version 1.1.03 were used for analysis. PFS and OS curves were created using the Kaplan–Meier Product-limit method. The median PFS and OS were obtained with a 95% CI. Cox proportional hazard model was used for the identifying factors affecting PFS and OS. A p-value of 0.05 or below was considered as significant. The factors considered were age, sex, subsite, ECOG PS, tobacco exposure and circulating endothelial cells.

### Sample size

#### Stage 1

Flexible-finite sample-based approach was adopted to select the circulating endothelial cells (CEC) interval boundaries and further to use optimum interval design. During the trial, we continuously updated the posterior estimates of the decline in CEC and clinical benefit rate and assigned patients to the most appropriate dose combination). The Bayesian adaptive screening design was used to simultaneously select among possible treatment dose combinations. The design is based on formulating the selection procedure as a Bayesian hypothesis testing problem in which the superiority of each treatment combination is equated to a single hypothesis. During the trial conduct, we used the current values of the posterior probabilities of all hypotheses to adaptively allocate patients to treatment combinations. The dose-finding decision was decided on the twin endpoints: the clinical benefit rate at 2 months and proportional decline in CEC at D8 of first cycle.

#### Stage 2

It was assumed that the 3-month progression-free survival (PFS) rate of a standard cancer therapy was 0.50 (H0). An improvement to 0.60 was considered clinically significant (H1). Assuming both the null and alternative PFS distributions follow an exponential distribution, the 1-sided type I error rate will be of 0.05 and type II error rate of 0.1. Prior assumption included a fixed enrollment rate of 3 patients per month. We made a stop at 3 months at the end of Stage 1. The library (“OptInterim”) was used to calculate the sample size. A 20% extra patient was taken into account for lost to follow up data. Hence a total of 76 (63 patients + 13 patients) sample size was found suitable to carry out the study for stage II with single arm drug therapy.

#### Role of funding source

The sponsors had no role in design, conduct, data collection, analysis, interpretation or on the decision to submit the manuscript for publication.

## Results

A total of 91 patients were recruited (15 in phase 1 & 76 in phase 2). The baseline characteristics among the whole study population is shown in Table 1.

**Table 1:** Baseline characteristics and previous treatment details.

Variable	Total–no. (%)
Age-yr	
Median	41
Range	(25–66)
Gender-no. (%)	
Male	84 (92.3)
Female	7 (7.7)
ECOG PS-no. (%)	
0	7 (7.7)
1	80 (87.9)
2	4 (4.4)
Smoking status-no. (%)	
Never smoker	61 (67)
Former or current smoker	30 (33)
Tobacco chewer-no. (%)	
Yes	74 (81.3)
No	17 (18.7)
Subsite-no. (%)	
Buccal mucosa	52 (57.1)
Anterior ⅔ tongue	25 (27.5)
Others^a^	14 (15.4)
Indication-no. (%)	
Early failure	4 (4.4)
Platinum insensitive disease	87 (95.6)
Sites of failure-no. (%)	
Locoregional-no. (%)	81 (89)
Distant	4 (4.4)
Both	6 (6.6)
Previous treatment intent	
Curative	75 (82.4)
Palliative	16 (17.6)
Lines of previous systemic treatment received	
1	67 (73.6)
2	19 (20.9)
>2	5 (5.5)
Previous chemotherapy-no. (%)^b^	
Platinum	87 (95.6)
Paclitaxel	20 (22)
Docetaxel	32 (35.2)
Cetuximab	1 (1.1)
Nimotuzumab	1 (1.1)
5 FU or Capecitabine	14 (15.4)
Previous radiation received-no. (%)	
Yes	48 (52.7)
No^c^	43 (47.3)
Time interval between last chemotherapy and failure-months	
Median	1
Range	0–6

FU - Fluorouracil.

a Other include floor of mouth, hard palate, alveolus, lip and retromolar trigone.

b Some patients had received more than 1 systemic therapy and hence the total sum might be more than the number of patients.

c The primary reason for not receiving palliative radiation were gross mandibular erosion in 28 patients, orocutaneous fistula in 13 patients, metastatic disease not eligible for radiation in 1 patient and patient refusal in 1 patient.

### Overall survival

**Fig. 1:**
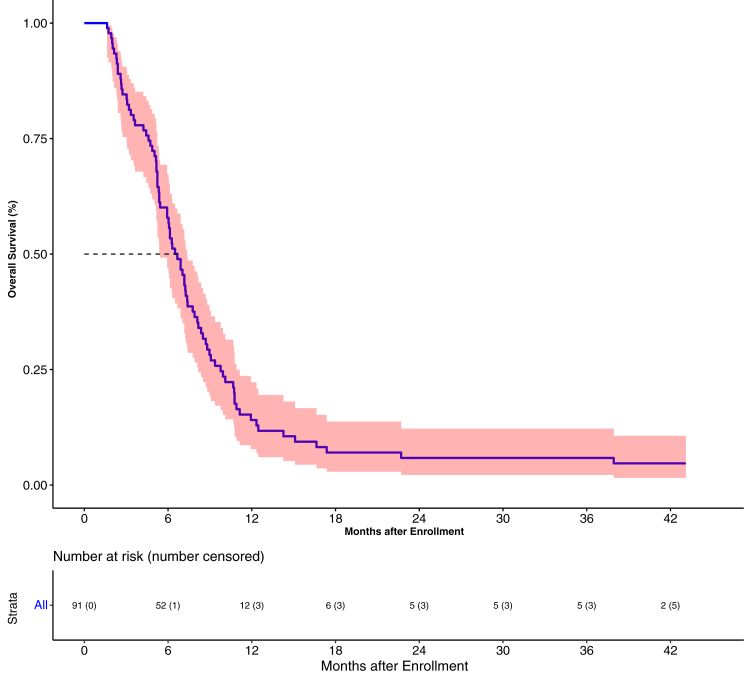
Overall survival graph.

The median follow-up was 41 months and 84 events of death had occurred. The median OS was 6.7 months (95% CI 5.4–7.4). The 1-year, 2-years and 3-year OS’ were 14.1% (95% CI 7.8–22.2), 5.9% (95% CI 2.2–12.2) and 5.9% (95% CI 2.2–12.2) respectively (Fig. 1). The only factor favorably impacting OS was the detection of circulating endothelial cells at baseline (HR = 0.46; 95% CI 0.28–0.75, P = 0.0020) (Table 2). The median OS in accordance in phase I was 5.23 months (95% CI 4.73–7.77) and the 1-year OS was 6.67% (95% CI 0.4–26) versus the corresponding value in phase II were 6.9 months (95% CI 6.03–8.1) and 1-year PFS was 15.6% (95% CI 8.35–24.9) (P = 0.11).

**Table 2:** Factors affecting overall survival.

Variable	Hazard ratio	95% CI of Hazard ratio	P-value
Age	0.992	0.9768–1.0216	0.5106
Gender - Male	1.083	0.4659–2.5655	0.856
Reference - Female			
ECOG PS 0-1	10.00996	0.462–2.145	0.991
Reference - ECOG PS 2			
Tobacco exposure absent	0.620	0.3329–1.1768	0.1439
Reference - Tobacco exposure present			
Subsite - Oral	1.5766	0.914–2.683	0.102
Reference - Subsite Non-Oral			
Circulating Endothelial Cells (CEC)	0.4659	0.280–0.753	0.0020

CI - Confidence Interval, ECOG PS - Eastern Cooperative Oncology Group Performance Status.

### Progression-free survival

**Fig. 2:**
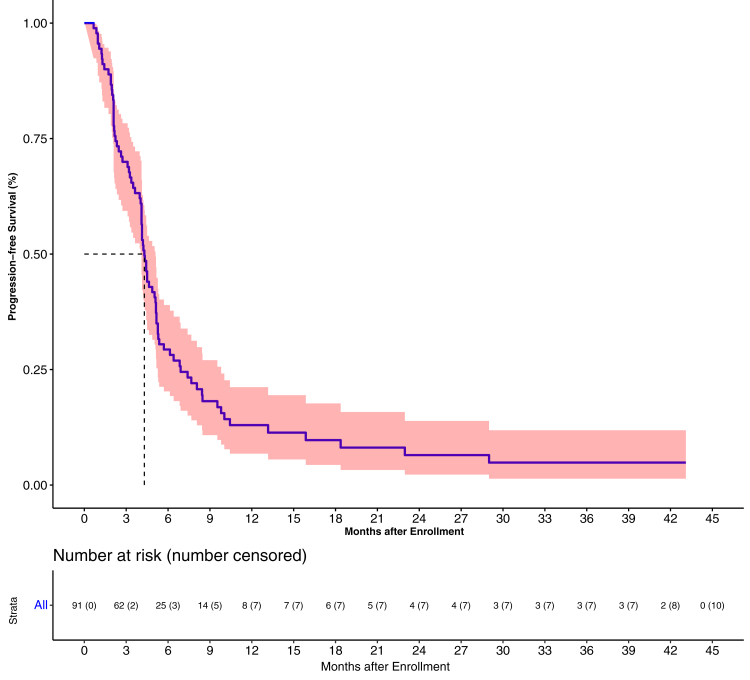
Progression free survival graph.

The median PFS was 4.3 months (95% CI 4.1–5.1) and the 1-year PFS was 13.0% (95% CI 6.8–21.2). The 2 and 3 year PFS was 6.5% (95% CI 2.3–13.9) and 4.9% (95% CI 1.4–11.9) (Fig. 2). The factors with a statistically significant impact on PFS were detection of circulating endothelial cells at baseline (HR = 0.48; 95% CI 0.30–0.78, P = 0.0020) and no tobacco exposure at baseline (HR = 0.51; 95% CI 0.27–0.94, P = 0.030) (Table 3). The median PFS in accordance in phase I was 4.10 months (95% CI 2.1–4.63) and the 1-year PFS was 6.67% (95% CI 0.4–26) versus the corresponding values in phase II were 4.43 months (95% CI 4.1–5.27) and 1-year PFS was 14.15% (95% CI 7.12–23.5) (P = 0.11).

**Table 3:** Factors affecting progression-free survival.

Variable	Hazard ratio	95% CI of Hazard ratio	P-value
Age	0.984	0.9659–1.009	0.203
Sex Gender - Male	1.252	0.5106–3.10098	0.6326
Reference - Female			
ECOG PS 0-1	1.4985	0.672–3.282	0.3328
Reference - ECOG PS 2			
Tobacco exposure absent	0.5105	0.272–0.9437	0.030
Reference - Tobacco exposure present			
Subsite - Oral	1.0546	0.6105–1.8108	0.872
Reference - Subsite Non-Oral			
Circulating Endothelial Cells (CEC)	0.480	0.30297–0.7875	0.0030

CI - Confidence Interval, ECOG PS - Eastern Cooperative Oncology Group Performance Status.

**Fig. 3:**
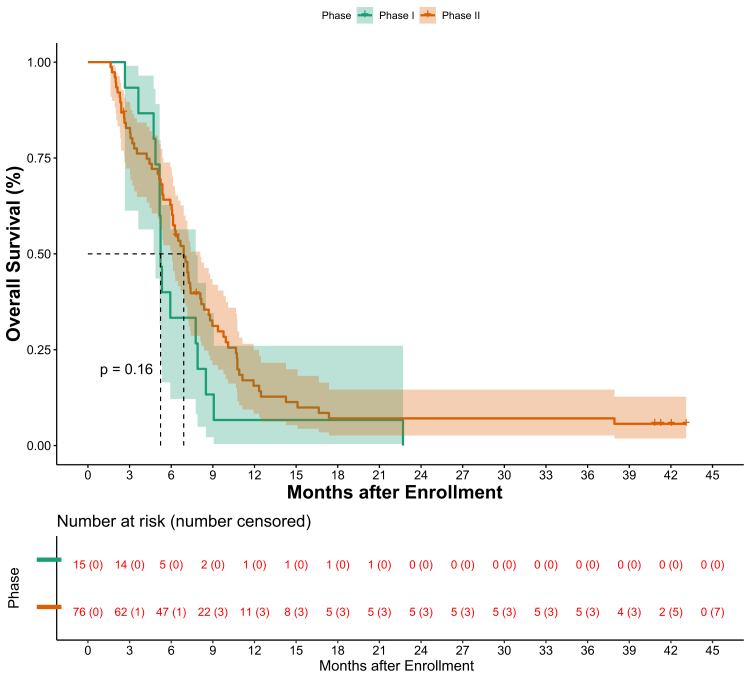
Figure depicting the overall survival in accordance with phase of the study.

**Fig. 4:**
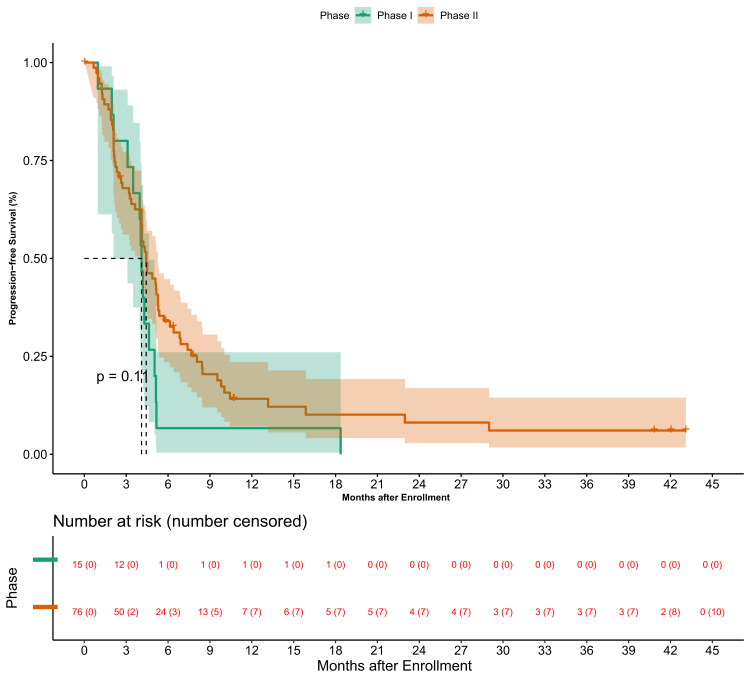
Figure depicting the progression free survival in accordance with phase of the study.

Overall survival by phase is depicted in Fig. 3. Progression-free survival by phase is depicted in Fig. 4.

### Adverse events

Data on outcomes and adverse events have been published in the early study.^13^ We have this information for a total of 88 patients. Common adverse events of any grade include fatigue (75 patients, 85.2%), rash (71 patients, 80.7%) and anemia (71 patients, 80.7%). 12 patients (13.6%) ended up requiring a dose reduction.

## Discussion

In our Phase I/II study, the long-term outcomes with the use of triple metronomic chemotherapy (TMC) at 1 year and 2 years was disappointing. In a study by Ferris RL et al.^4^; the 2 year survival rate in platinum-refractory, recurrent metastatic head and neck cancer was 16.9%. In our study, the 2 year survival was 5.9%. This was in spite of having similar median progression free survival and overall survival. This suggests that the outcomes with immunotherapy are sustainable but this is not the case with metronomic regimens. The inferior outcomes can also be due to the differential profile of the patients. All the patients included in our study had primary in the oral cavity this is in contrast to what was seen in the study reported by Ferris et al. where primary was in the oral cavity in below 50% of the patients. Outcomes from oral cavity cancers are generally worse compared to other head and neck cancer sites. Further in developing countries, patients tend to report very late to cancer centers and with high tumor burden as compared to the high income countries and this leads to dismal outcomes.^14^

Metronomic chemotherapy has been a boon for low and middle income countries especially in head and neck cancers. Post the pivotal phase III study by Patil V et al.^14^ where oral metronomic chemotherapy showed efficacy over single agent cisplatin with median overall survival in the intention-to-treat analysis population of 7.5 months (IQR 4.6–12.6) in the oral metronomic chemotherapy group compared with 6.1 months (3.2–9.6) in the intravenous cisplatin group (unadjusted HR for death 0.77 [95% CI 0.62–0.97, p = 0.026]); multiple centers in India have started using oral metronomic regimen. Experience from South India,^15^^,^^16^ North India,^17^ Western India^18^ and East India^19^ have published their experiences with oral metronomic regimen. The median OS in all these studies ranged between 5 and 7 months. All these studies have stressed on the fact that there were lower adverse events and better response rates observed with metronomic regimen. However all of them except one^19^ are retrospective and none had reported long term outcomes. Hence, this is the importance of our study which is a large prospective study and has reported long term outcomes.

In our study, we tried to identify factors that impact progression-free survival and overall survival in patients with early-failure oral cancer on triple metronomic chemotherapy. The factors with a statistically significant impact on PFS were detection of circulating endothelial cells and no tobacco exposure at baseline. The factor that had an impact on OS was detection of circulating endothelial cells at baseline. Thus suggesting that detection of circulating endothelial cells can be a biomarker for prediction of outcomes with metronomic chemotherapy. This is also a hypothesis assuring finding, as the action of metronomic chemotherapy is postulated to be on circulating endothelial cells^20^^,^^21^. Further research in this area might enable us to identify the patients who may benefit or not benefit from metronomic chemotherapy.

There are certain limitations to our study. This is a single center, non-randomized study and only oral cavity cancer patients were included in this study while the other sites of head and neck cancer were excluded. A strength of our study is that by focussing on patients with cancer of the oral cavity, we've accrued data on a highly prevalent site of cancer for the Indian population. Relative to other sites, oral cavity cancer patients have poor outcomes amongst head and neck cancers.^22^^,^^23^ National Comprehensive Cancer Network (NCCN) recommends pembrolizumab in recurrent, metastatic head and neck cancer. However, affordability is a challenge in cancer patients from low and middle-income countries. Thus, there is an unmet need to have alternative, cheaper treatment options.^24^ There are reports showing benefit of low dose immunotherapy in head and neck cancer patients.^25^ This was the rationale behind further evaluating long-term outcomes with TMC.

There is a need in the future to have a randomized study comparing the low dose immunotherapy alone versus combination of triple metronomic chemotherapy with low dose immunotherapy in platinum-refractory head and neck cancer.

The long-term outcomes with triple oral metronomic chemotherapy with erlotinib, methotrexate and celecoxib are unsatisfactory. Detection of circulating endothelial cells at baseline is a biomarker predicting efficacy of this therapy. The factors with a statistically significant impact on PFS were detection of circulating endothelial cells and no tobacco exposure at baseline. The factor that had an impact on OS was detection of circulating endothelial cells at baseline.

## Contributors

The authors confirm contribution to the paper as follows:

Study conception and design: VP, KP.

Data collection: All authors.

Analysis and interpretation of results: All authors.

Draft manuscript preparation: All authors.

All authors reviewed the results and approved the final version of the manuscript. All authors vouch for the accuracy and completeness of the data, analyses and for the fidelity of the study to the study protocol.

## Data sharing statement

De-identified data may be shared on a case-by-case basis upon reasonable requests to the corresponding author for a period of 5 years.

## Declaration of interests

**Vijay Maruti Patil receivedfundingfrom**10.13039/100004325AstraZeneca (Inst); 10.13039/501100003769Eisai Germany (Inst); INTAS (Inst); 10.13039/100004331Johnson & Johnson/Janssen (Inst); NATCO Pharma (Inst); 10.13039/100004336Novartis (Inst).

**Vanita Noronha receivedfundingfrom**10.13039/100002429Amgen (Inst); 10.13039/100004325AstraZeneca (Inst); Dr. Reddy's Laboratories (Inst); INTAS (Inst); 10.13039/100004339Sanofi/Aventis (Inst).

**Kumar Prabhash receivedfundingfrom**Alkem Laboratories (Inst); BDR Pharmaceutics (Inst); 10.13039/100007777Biocon (Inst); Dr. Reddy's Laboratories (Inst); 10.13039/100017530Fresenius Kabi (Inst); NATCO Pharma (Inst); 10.13039/100004337Roche (Inst).

## Appendix A Supplementary data

Triple metronomic_ Final protocol
