# Supplementary material for: Long term outcomes of phase I/II study of palliative triple metronomic chemotherapy in platinum-refractory/early failure oral cancer
Source: Lancet Reg Health Southeast Asia. 2023 Mar 1;12:100143. doi: 10.1016/j.lansea.2023.100143 (PMC10306045; doi:10.1016/j.lansea.2023.100143)
Supplement: Triple metronomic_ Final protocol [file mmc1.pdf]

Clinical trial protocol

Title : “Stage I/II study of oral metronomic methotrexate with celecoxib and erlotinib as palliative chemotherapy in oral cancer patients”

Protocol version 3.0
Dated : 4th January 2016

PI : Dr Vijay M patil
Co-I
Vanita Noronha
Amit Joshi
Manoj Mahimkar
Anuradha Chougule
Rajiv Kaushal
Anand Patil
Kumar Prabhash

1 Table of contents

1 Table of contents
2 Summary of proposed research
3 Concept sheet
4 Background
    4.1 Palliative chemotherapy in oral cancers
    4.2 Metronomic chemotherapy (celecoxib and oral methotrexate) in Head and Neck cancers
    4.3 Role of erlotinib in Head and neck cancers
    4.4 Rationale for combination of erlotinib and celecoxib with methotrexate (MTX)
    4.5 Metronomic dose chemosensitivity biological mechanisms: Antiangiogenic
    4.6 Metronomic dose chemosensitivity biological mechanisms: Immune system modulation
    4.7 Need for novel design for finding optimal biological and effective metronomic dose of oral methotrexate
    4.8 PK-PD (Pharmacokinetics- Pharmacodynamics) study of Methotrexate
5 Rationale for the study
6 Hypothesis
7 Objectives
    7.1 Primary objective
    7.2 Secondary objective (both stages)
    7.3 Tertiary objective (both stages)
8 Study Design
9 Study Setting
10 Eligibility Criteria
    10.1 Inclusion Criteria
    10.2 Exclusion Criteria
11 Interventions
    11.1 Prerequisite for administration of metronomic therapy
    11.2 Metronomic chemotherapy administration
        Missing dose
        Delivery setting
        General Concomitant Medications and Supportive Care Guidelines
        Duration of Therapy
        Followup
    11.3 Removal of Patients from Protocol
    11.4 Toxicities and Dosing Delays / Dose Modifications
        Hematological Toxicities
        Non Hematological Toxicity
            Nausea & Vomiting
            Nephrotoxicity

Hepatic Dysfunction
Potential Drug Interactions
11.5 Compliance Criteria
11.6 Imaging Plan
12 Study procedure
12.1 Clinical Intervention
12.2 Laboratory interventions
Sample collection:
Flow cytometry procedure
Timeline for doing flow cytometry
Procedure
Processing of sample
Interpretation of results
ELISA
Procedure
Processing of sample
Tissue based markers (IHC or microarray)
Analysis of markers of hypoxia and angiogenic markers in tumor tissues by IHC
Detection of HPV
Cell proliferation markers by IHC
Apoptosis markers by IHC
Inflammation markers by IHC
Microarray based Gene Expression Analysis
Microarray based global miRNA profiling of serum.
Need for multiple biopsies
13 Time and Events Table
14 Study timeline
15 Sample size calculation
16 Outcome measures
16.1 Primary outcome :
16.2 Secondary and tertiary outcomes:
17 Data Collection Methods
18 Data Management
19 Quality assurance
20 Monitoring
20.1 Data Monitoring
20.2 Interim Analysis
21 Harms
22 Auditing
23 Ethics and Dissemination
23.1 Ethics Approval
23.2 Protocol amendments
23.3 Consent / Assent

23.4 Confidentiality
23.5 Access to Data
23.6 Ancillary and post trial care
24 Dissemination Policy
25 Feasibility
26 Funding
27 Risks
28 References

2 Summary of proposed research

Problem statement & rationale : A combination of platinum based chemotherapy with cetuximab is the recommended palliative chemotherapy regimen in head neck cancers., though less than 1% of patients can afford the treatment in developing countries. As a significantly cheaper alternative, we have designed an oral metronomic chemotherapy (MCT) combination of methotrexate and celecoxib. In a randomized study, we showed a survival advantage along with with lower side effects, better QOL and fewer hospital visits compared to single agent cisplatin. One third of failures in head and neck cancers are seen within 6 months, and such patients have median survival of less than 3 months. This MCT was studied in these patients with median expected survival of 4 months (sent for publication). Despite the impressive initial efficacy of MCT, resistance usually develops in 3-6 months. Overexpression of multidrug transporters (Abcc2, Abcc3, and Abcg2) may be one of the reason behind the development of resistance to methotrexate used in MCT. Of these, Abcg2 is active in head and neck cancers and over-expressed in 50-60% of patients. Erlotinib (E) an oral tyrosine kinase inhibitor can inhibit Abcg2 mediated drug efflux. In our experience erlotinib when used after failure of cisplatin or MCT results in a promising median survival of 5.06 months.(send for publication). Upfront combination of MCT with erlotinib (MCT-E) studied by the author resulted in a 6 month survival of 80%.(accepted for publication). However these results need confirmation in a large study, along with translational research to identify the mechanism of action, resistance and biomarkers for efficacy prediction.

Drugs like methotrexate have an anti-angiogenic (metronomic) action when administered at ⅓ to 1/10 of maximum tolerable dose. We had selected a dose of 15 mg/m2 for our MCT regimen as it was 1/3rd of the maximum tolerated dose dose (MTD) . Initial preclinical data suggests that there is a dose range in which this anti-angiogenic action is seen and it is unknown if a lower dose will result in better anti-angiogenic activity. As traditional dose escalation based phase I designs are inappropriate for testing this hypothesis, we have designed a study which would incorporate clinical response and a laboratory biomarker, serum circulating endothelial cells (CECs) to determine the most biologically effective metronomic dose of methotrexate. We aim to utilize CECs as a surrogate serum marker for tumor related angiogenesis for this study.

Study design and objectives: This would be a prospective observational single arm study conducted in 2 stages. The primary objective of the first-stage would be to identify the optimal biological effective dose (OBD) of methotrexate in combination with celecoxib (200 mg PO BD) and erlotinib (150 mg PO OD).

The primary objectives of the second-stage would be to estimate the median progression free survival (PFS) and overall survival (OS) with the combination of methotrexate used at the OBD with celecoxib and erlotinib.

The secondary objectives would be to evaluate the changes in the profile of serum biomarkers (VEGF, VEGFR1-3, Tie-2, Angiopoietin -1-2, Thrombospondin 1, Vasoinhibin, Angiostatin, Endostatin ,IL-2,CECs) tissue angiogenic biomarkers (Thymidine phosphorylase, HIF- I-alpha,

DLL, SPARC) and tissue tumor immunity related biomarkers (Foxp3 +ve T reg cells) during the course of MCT. To study the PK/PD of low dose methotrexate.
The tertiary objective would be to study gene expression changes in the tumor tissue during MCT to identify responders and mechanisms of resistance.

Study procedures (clinical): The study population would include patients with chemoresistant oral cancers, fit for MCT-E. In stage 1, a dose de escalation design with continuous reassessment model would be used to identify OBD of methotrexate. The disease control rate at 2 months and the proportional decline in CECs would be used for decision making regarding de escalation. Around 20-30 patients would be required for stage 1. In the second stage the combination of MCT-E would be administered in 76 patients. These patients would be followed at 8th day then subsequently every 30th day till death.
Disease progression would be defined as per RECIST criteria version 1.1. Serum and tissue biopsy would be collected in all patients at baseline prior to the start of MCT-E, at day 8 and at progression. In addition serum samples will be collected at monthly intervals till progression for serial assays of serum biomarkers.

Study procedures (laboratory): CECs would be measured in serum by flow cytometry. The other serum angiogenesis related markers would be quantified by ELISA. The tissue tumor immunity and angiogenesis markers would be measured by immunohistochemistry. Microarray would be used to profile the gene expression profile in the tumor specimens. PK/PD would be studied by plasma methotrexate drug level analysis.

3 Concept sheet

Primary Registry	Clinical Trial Registry of India (CTRI)
Contact for Scientific Queries	Dr Vijay M Patil, DM, Department of Medical Oncology, Tata Memorial Centre<br>Email: vijaypgi@gmail.com
Public Title	Study to find the recommended metronomic dose of oral methotrexate in palliative chemotherapy and its effect in oral cavity cancers
Scientific Title	**“Stage I/II study of oral metronomic methotrexate with celecoxib and erlotinib as palliative chemotherapy in oral cancer patients”**
Countries of Recruitment	India
Health Condition Studied	Squamous cell cancers of head and neck region warranting palliative chemotherapy
Interventions	Weekly oral chemotherapy with:<br>De escalating dose of oral methotrexate given weekly D1<br>1. Dose Level 0: 15 mg/m^2<br>2. Dose Level I: 12 mg/m^2<br>3. Dose Level II: 09 mg/m^2<br>4. Dose Level III: 06 mg/m^2<br>5. Dose Level IV: 03 mg/m^2<br>Fixed dose of erlotinib 150 mg OD<br>Fixed dose of celecoxib 200 mg BD
Aim	Stage 1 : To identify the optimal biological effective dose (OBD) of methotrexate in combination with celecoxib (200 mg PO BD) and erlotinib (150 mg PO OD)<br><br>Stage 2 : To estimate the median PFS in head and neck cancer patients warranting palliative chemotherapy with the combination of celecoxib 200 mg BD, erlotinib 150 mg OD and oral weekly methotrexate
Objectives	Primary (Stage 1)<br>1. To find out the optimal biological and clinically effective metronomic dose of weekly oral methotrexate when given in combination with celecoxib (

<table>
<tr><td></td><td>200 mg BID PO Daily) and erlotinib (150 mg OD daily)
Primary (Stage 2) :
1. To find out the median PFS in Head and neck cancer patients warranting palliative chemotherapy with the combination of celecoxib 200 mg BD, erlotinib 150 mg OD and oral weekly methotrexate in biologically optimal doses
Secondary (both stages):
1. Frequency of maximum grade of toxicity (hematological and non hematological) in accordance with CTCAE version 4.03 with the combination metronomic therapy
2. Pattern of change in biomarkers according to disease control
a. Serum angiogenic biomarkers : VEGF, VEGFR1,VEGFR2,VEGFR3, Tie-2, Angiopoietin -1, Angiopoietin -2, Thrombospondin 1, Vasoinhibin, angiostatin, endostatin ,IL-2, CECs and Endothelial progenitor cells
b. Tumor tissue angiogenic marker : Thymidine phosphorylase, Hypoxia inducible factor I-alpha, DLL, SPARC
c. Tissue tumor immunity related biomarkers :Foxp3 +ve T reg cells
3. To develop Pharmacokinetics-Pharmacodynamics (PK-PD) Model of methotrexate using CECs and serum biomarkers as PD endpoints
Tertiary (both stages)
1. To study gene expression changes in the tumor tissue during MCT to identify responders and mechanisms of resistance.</td></tr>
<tr><td>Population</td><td>Inclusion Criteria
1. Histologically proven squamous cell cancers of oral cavity with at least one measurable lesion.
2. Age > 18 years
3. ECOG PS 0 - 2
4. No uncontrolled comorbidities
5. Not affording cetuximab based chemotherapy
Exclusion Criteria
1. Progression free survival or disease free interval less than 6 months
2. Primary sites of malignancy in major salivary gland or nasopharynx or skin would be excluded
3. Patients receiving methotrexate for other indications will be excluded
4. Patients who had received long term Cox-2 inhibitors (more than 3 month continuous usage) will be excluded
5. Patients with Grade 3 and above CTCAE version 4.03 QTc interval prolongation will be excluded
6. Patients who are pregnant, who are HIV positive and those with psychiatric</td></tr>
</table>

	illness will be excluded
Study Type	Nonrandomized open label dose de escalation study followed by single arm open label nonrandomized observational study
Study procedure	**Intervention** : Patients would receive the intended oral therapy<br>These patients would be monitored for response and toxicity (CTCAE version 4.02) at D8 and D30. Following which they would be assessed every month.<br>**Biomarker** : All patients would undergo serum biomarker investigations at least 1 day before administration of oral therapy, then at at D8, D30 and for next 3 months<br>**Biopsy** : Done at baseline, then at at D8 and at progression.<br>**Response (Radiology)** : Done at baseline and then at 1 monthly intervals
Sample Size	Around 20-30 patients would be required for stage 1. In the second stage the combination of MCT-E would be administered in 76 patients.
Date of first enrollment	Post IRB/IEC and grants approval.
Feasibility	We expect to complete accrual for the stage 1 study assuming the maximum number of patients within 24 months. For stage 2 study with maximum accrual in another 24 months.
Significance	1. This would be the first study to systematically determine the optimal biologically and clinically effective metronomic dose of methotrexate<br>2. Evolution in serum and tissue biomarker levels during metronomic chemotherapy and their relationship to disease control would be identified<br>3. Gene expression markers predicting for response would be identified<br>4. Gene expression changes at progression would be identified<br>5. If MCT-E improves median PFS > 3 months then a potential new option would be created in platinum resistant oral cancers
Funding	Intramural and Extramural funding to be sought
Risks	Risks associated with the protocol include usual side effects of erlotinib (anorexia, fatigue, diarrhea and rash), celecoxib (gastritis) and low dose methotrexate (oral mucositis)

4 Background

4.1 Palliative chemotherapy in oral cancers

Palliative chemotherapy in head and neck cancers has evolved over three and a half decades. In a small study, use of single agent chemotherapy using cisplatin improved survival over best supportive care.[1] Single agent methotrexate and cisplatin were found to have similar efficacy and were in common use.[2] Use of combination chemotherapy was then tested in subsequent decades. Both 2 drug combinations and 3 drug combinations have been used. However unfortunately none of these combinations provided a survival benefit over single agent platinum therapies with median survival observed to be around 6 - 8 months.[2, 3] However as these combinations provided a higher response rate and longer duration of progression free survival, they were reserved for young, fit and symptomatic patients.

Addition of cetuximab to platinum-based chemotherapy with fluorouracil (platinum–fluorouracil) prolonged the median overall survival from 7.4 months in the chemotherapy-alone group to 10.1 months in the group that received chemotherapy plus cetuximab (hazard ratio for death, 0.80; 95% confidence interval, 0.64 to 0.99; P=0.04).[4] Since then several guidelines consider this regimen as the standard of care for palliative chemotherapy regimen in Head and neck cancers. However patients who failed within 1 month of surgery/ radiation or within 6 months of platinum based systemic therapy were excluded in the landmark cetuximab study (EXTREME). These patients were considered as having biologically aggressive disease and a platinum insensitive disease. At present there is no recommended treatment for these patients. In fact the NCCN ,ESMO and ICMR guidelines never have addressed these patients separately.[5–7] There are a few phase 2 studies showing benefit of cetuximab single agent and in combination with chemotherapy in patients progressing on platinum.[8, 9] Hence it can be said that cetuximab may be considered as an sole effective treatment option in this situation.

In developing countries like India access to cetuximab is an issue. In our own experience less than 1% of our patients can afford it.Similar situation prevails in Africa too.[10] In fact as oral cancers are seen in low socioeconomic strata affordability of intravenous chemotherapy is also questionable.[11] Its a sad fact that the maximum benefit of cetuximab was seen in oral cancer patients but oral cancer patients warranting it in low and middle income countries can't afford it.[4]There is a dire need for development of cost effective treatment options in these patients.

4.2 Metronomic chemotherapy (celecoxib and oral methotrexate) in Head and Neck cancers

Metronomic chemotherapy has been developed as a cost effective palliative chemotherapy option in head and neck cancers. Recently we conducted a randomized study of metronomic combination of celecoxib (200 mg BD) and oral weekly methotrexate (15 mg/m2 weekly). In this study metronomic chemotherapy was compared against cisplatin chemotherapy. Both the median progression free survival and overall survival were significantly better than cisplatin.. Patients in the metronomic arm had significantly longer PFS (median 101 days, 95% CI: 58.2–143.7 days) compared to the cisplatin arm (median 66 days, 95% CI; 55.8–76.1 days) (p = 0.014). The overall survival (OS) was also increased significantly in the metronomic arm (median 249 days, 95% CI: 222.5–275.5 days) compared to the cisplatin arm (median 152 days, 95% CI: 104.2–199.8 days) (p = 0.02).[12] This is probably the only trial except EXTREME study (cetuximab study) which showed a survival benefit of a chemotherapy over cisplatin. As discussed in above section previous studies of combination chemotherapies had failed to show a survival benefit over cisplatin. Additional these results of metronomic chemotherapy were achieved with much lower side effects and lesser amount of hospital visits. This is an oral chemotherapy regimen and the cost is around 1000-2000 INR per month as opposed to cost of combination chemotherapy (Paclitaxel+ carboplatin) been around 15,000-20,000 INR month and cost of cetuximab been nearly 4.0 lakh INR / month. Metronomic chemotherapy hence seems an affordable, accessible, safe and efficacious regimen in oral cancers. However the cohort of patients used in our metronomic randomized study had to be either chemonaive or need to have a failure free period of 3 months or more.

In patients who had failed within 1 month of surgery/ radiation or within 6 months of platinum based systemic therapy who couldn't afford cetuximab we offer metronomic chemotherapy at present. In our retrospective experience (data sent for publication) in oral cancers when such patients with early failures are subjected to metronomic chemotherapy the median survivals are much below expectations. The median estimated overall survival was 110 days (95% CI 85-134 days). There is an urgent unmet need to modify metronomic schedule so that such likely chemoresistant patients can be effectively treated with a cost effective option.

4.3 Role of erlotinib in Head and neck cancers

Head and neck cancers have high EGFR expression is seen in around 95% of patients.[13] Oral TKI like erlotinib or gefitinib have shown to have promising activity in head and neck cancers. In a study by Thomas et al in nact setting , erlotinib was given for a median of 20 days. At the time of surgery, tumor shrinkage was observed in nine patients (29%).[14] Even in palliative care setting in our own experience (data send for publication) second line erlotinib after first line chemotherapy failure provides a median estimated PFS and OS of 110 days (95%CI 61-175 days) and 156 days (95%CI 126-185 days) respectively. Reason for failure to erlotinib is development of resistance. One of the mechanism of resistance to erlotinib is COX-2 mediated angiogenesis.[15]

Celecoxib is an COX-2 inhibitor.[16] Head and neck cancers do have an overexpression of COX-2. Its has been used with erlotinib in chemoprevention studies in Head and neck cancers.[17, 18] In NACT setting too Gross et al studied erlotinib alone and in combination with sulindac (COX inhibitor). Brief treatment with erlotinib significantly decreased proliferation in HNSCC, with additive effect from sulindac.[19] They suggested that efficacy studies of dual EGFR–COX inhibition are justified. Erlotinib in combination with COX-2 has been studied in recurrent head and neck cancers with reradiation. With radiation a dose of erlotinib of 150 mg , a dose of celecoxib up till 400 mg per day was well tolerated. It also lead to promising 1-year locoregional control, progression-free survival, and overall survival rates of 60%, 37%, and 55%, respectively.[20] Combination of erlotinib to the metronomic combination of methotrexate and celecoxib would be an interesting prospect.

4.4 Rationale for combination of erlotinib and celecoxib with methotrexate (MTX)

Combination of erlotinib and celecoxib has a strong biological rationale. Metronomic chemotherapy is presumed to act by antiangiogenesis. Hence combination of metronomic with erlotinib would overcome one of the mechanism of resistance of erlotinib.[15] Abcc2, Abcc3, and Abcg2 are multidrug transporters which together mediate the rapid elimination of MTX and 7OH-MTX from the cell.[21] Abcg2 transporter is expressed in head and neck cancer s in around

50-65% of patients. its considered an unfavourable prognostic factor.[22] Erlotinib reverses ABCB1- and ABCG2-mediated MDR in cancer cells through direct inhibition of the drug efflux function of ABCB1 and ABCG2.[23] Hence combination of erlotinib and metronomic chemotherapy is a logical step and may lead to improvement in outcomes.

The use of this regimen on compassionate basis in poor risk head and neck cancers has been described by VP (author) in his previous institute.(data accepted for publication). In a small prospective study in pretreated patients this combination had a response rate of 46.7% and had a promising median PFS of 148 days (95%CI 95.47-200.52 days). The study met its primary endpoint of improving median PFS over 120 days. Upfront combination of MCT with erlotinib (MCT-E) studied by the author resulted in a 6 month survival of 80%.(accepted for publication). These results need confirmation in a large study, with translational research to identify the mechanism of action, resistance and biomarkers for efficacy prediction.

4.5 Metronomic dose chemosensitivity biological mechanisms: Antiangiogenic

Angiogenesis is a hallmark of Cancer.[24] Metronomic chemotherapy predominant mechanism of action has been considered as antiangiogenic. [25] The antiangiogenic effect of any chemotherapy modality depends upon the balance it restores between proangiogenic and antiangiogenic factors.[26] Table 1 list details of angiogenic and proangiogenic factors which are implicated in carcinogenesis. In addition it also gives the known effect of metronomic scheduling of chemotherapy on each of these factors and method of measurement. The predominant effect of MCT on angiogenesis is mediated via a decrement in endothelial cells and its progenitors.[27–30] Decrement in proportion circulating endothelial cells seems to be influenced by the scheduling of chemotherapy dose.[25, 31] Maximum tolerated doses of chemotherapy are associated with a apoptosis of endothelial cells however post this phenomenon a rise in circulating endothelial cells is observed. As opposed to this low dose metronomic scheduling is associated with apoptosis of endothelial cells and it also inhibits mobilization of endothelial progenitor cells.[32] The most biologically effective metronomic dose of a given drug would lead to maximum suppression of circulating endothelial cells.[29, 33]

Factor	Effect on angiogenesis	Known effect of chemotherapy metronomic schedule
Endothelial progenitor cells	Pro	Decrement or stable[31]
Serum VEGF	Pro	Decrement or stable[34]
VEGFR 1,2,3	Pro	No effect[35]
PDGFR-beta	Pro	-
Thymidine Phosphorylase	Pro	- (it has been associated with response to metronomic doses of 5 FU)[36]
Tie-2 , Angiopoietin 1-2	Anti	-
Basic fibroblast growth factor	Pro (implicated in resistance)[27]	-
Hypoxia inducible factor 1 alpha	Anti	Decrement[37]
Thrombospondin 1	Anti	Increment in levels[38, 39]
SPARC	Anti	- (Over expression may be predictive of outcome)[40]
DLL4 ligand	Pro	-
IL-8	Pro	-
Angiostatin,Endostatin,Vasohibin	Anti	-
CAIX, Plasminogen activator inhibitor-1, Osteopontin	Pro	-

Table 1 : Pro & antiangiogenic factors

4.6 Metronomic dose chemosensitivity biological mechanisms: Immune system modulation

In addition to having antiangiogenic action metronomic chemotherapy also has anti tumor-immune effects. Metronomic chemotherapy decreases the immunosuppressive effect of

Tumor on host immune system.[41, 42] The major 2 ways by which metronomic chemotherapy helps in restoration of antitumor immune effect are

1. Inhibition of tumor tolerance inducing cells
    i. Treg (T-regulatory) cells
    ii. MDSC (Myeloid derived suppressor cells)
2. Improvement of antitumor response
3. Increased expression of tumor associated antigens
4. Enhancement in dendritic cell functioning

The contribution of these antitumor immune effects of metronomic towards its disease control in lights of its angiogenic properties are not known. FOXp3+Treg cells can be measured in tumor tissue by IHC.

4.7 Need for novel design for finding optimal biological and effective metronomic dose of oral methotrexate

Metronomic administration is actually a schedule. Same drugs when administered in maximum tolerable dose (MTD) would have different action and the same drugs when administered in 1/10 th to ⅓ rd of doses of MTD would have different mechanism of action.[24, 43] This phenomenon is seen in multiple chemotherapeutic drugs like paclitxael, cyclophosphamide, vinca alkaloids and in methotrexate.[24, 43]

The doses selected for metronomic studies have been arbitrary. Doses of 10-33% of MTD have been selected. The dose of methotrexate in Head and neck cancer metronomic was also selected in a similar way. This approach has been criticised by Lam et al. He stressed in his review on having a rationale design for finding an optimal dose of metronomic schedule.[44]

Traditional optimal dose finding designs of are based on tolerance to chemotherapy. Doses which lead to acceptable dose limiting toxicities are selected hence these chemotherapy regimes are labelled as maximum tolerable dose (MTD) chemotherapy The issue in metronomic schedules is that toxicity is not a concern. In our own experience with methotrexate and celecoxib only 18.9% patients had grade 3-4 adverse events (all inclusive) as opposed to 31.4% in cisplatin arm. Not only that toxicity is not a concern but the metronomic effect of a drug is supposed to happen at much lower doses than the MTD hence toxicity as used in traditional dose finding design cannot be used as an parameter for optimal dosing of an metronomic schedule. (Figure 1)

Dose de escalation strategies have been suggested by Emmenegger et al for optimal dosing of antiangiogenic drugs.[45] Figure 1 also clarifies why de escalation may be one of the best methods for optimal dosing of metronomic chemotherapy. As can be seen when the dose level decreases beyond a certain dose level the drug starts to act as an antiangiogenic agent. This property remains upto a certain low dose level and then again it starts disappearing. This window of antiangiogenic property is labelled as antiangiogenic window. This antiangiogenic window was described by Bocci et al and subsequently has been confirmed in other studies.[46]
Shaked et al used 4 distinct metronomic chemotherapy regimens in 4 different preclinical tumor models, they established the OBD (optimal biological dose) by determining the maximum efficacy associated with minimum or no toxicity. Subsequently they found each OBD to be strikingly correlating with the maximum reduction in viable peripheral blood circulating vascular endothelial growth factor receptor 2-positive (VEGFR-2+) endothelial precursors (CEPs). Circulating endothelial cell is a surrogate marker for antiangiogenic property of metronomic chemotherapy.[33]The antiangiogenic peak described in figure 1 is associated with maximum inhibition of circulating endothelial cells.[29, 33, 47, 48] Improvement in survival with metronomic schedules and its correlation with CEC/CEPs are described in literature.[47, 49, 50]

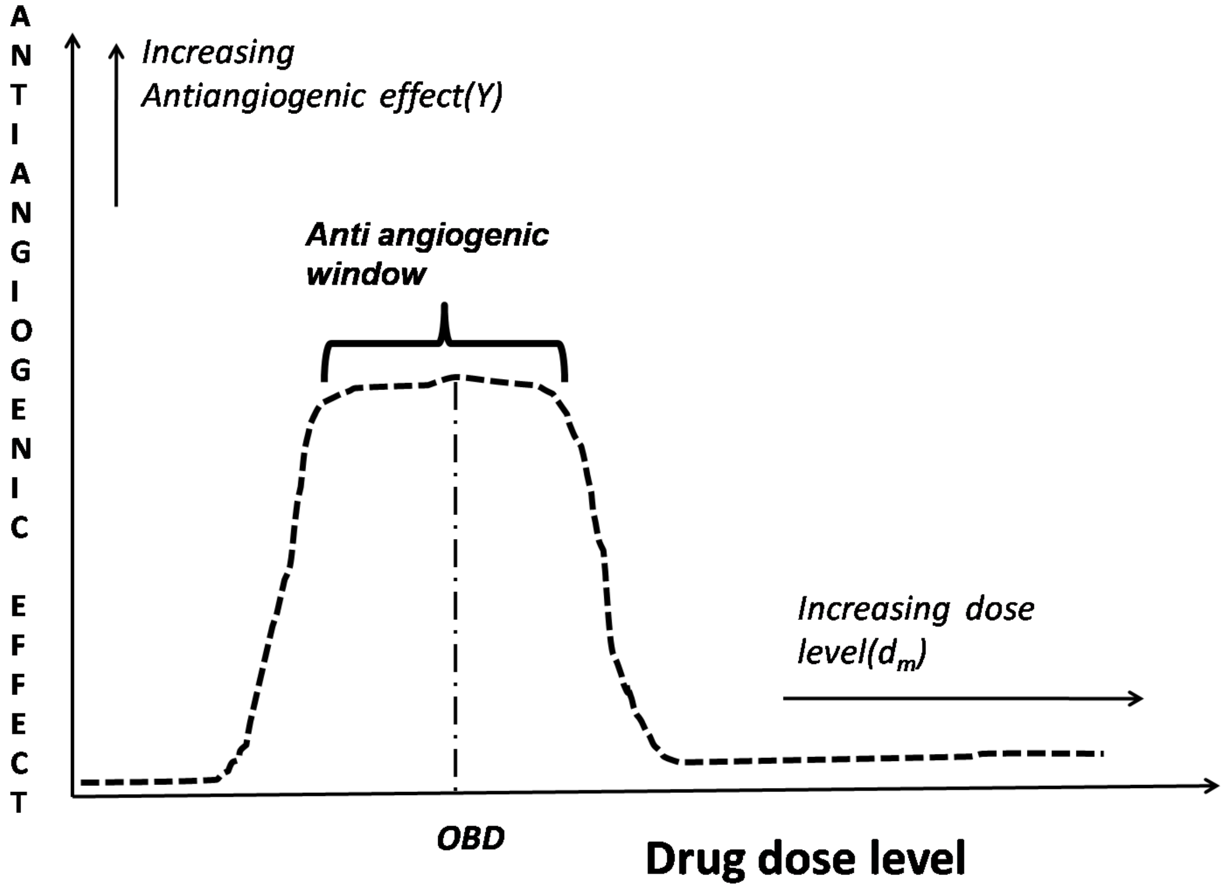

Figure 1 : As the drug dose decreases the antiangiogenic property of the drug starts increasing at a certain dose level, with further dose de escalation it would further increase however after a certain level of dose de escalation it would then start decreasing. Adapt from Bhattacharjee et al (unpublished data)

In de escalation strategies the starting dose level is selected on the basis of safety data. The response or endpoint of interest is documented for patients at this starting dose level (Dose level 0). Subsequently patients are treated at a lower dose level (Dose level -1). The response or the endpoint of interest is seen and compared to the starting dose level. Depending upon the response the decision is taken whether to continue de escalation or to stop. This design has been used for revealing the optimal dose of bevacizumab.[45] This design is very suitable for

metronomic studies. An modification of this design with bayesian approach and CRM (continuous reassessment model) modelling has been developed and validated by Bhattacharjee et al. (Data sent for publication) It has been validated through data simulation of docetaxel patients were response rate and biomarker was used for decision making. This model takes into account 2 things, disease control (clinical factor) and proportional decline in circulating endothelial cells (biological factor). In the model proposed by Bhattacharjee et al rather than waiting for 3 patients at each dose level, bayesian approach with CRM modelling enables the users to use lesser number of patients and achieve the results early. In this design the efficacy details of each patient are feed into the model and then the model would predict whether the investigators needs to continue at the same dose level or de escalate the doses. The decision rules for the model can be seen in figure 2. This model uses both factors response to MCT and proportional decline in circulating endothelial cells for decision making. Interestingly though response has been used as primary deciding endpoint. In most of the scenarios especially in oral cancers metronomic chemotherapy leads to disease stabilization. In such cases using disease control rate as an criteria for metronomic chemotherapy seems more suitable. Disease control rate is an combination of complete response, partial response and disease stabilization. The variation in CEC measurement is to the tune of 10% or below hence we have used a proportional decline in CEC level of > 10% as a deciding factor.

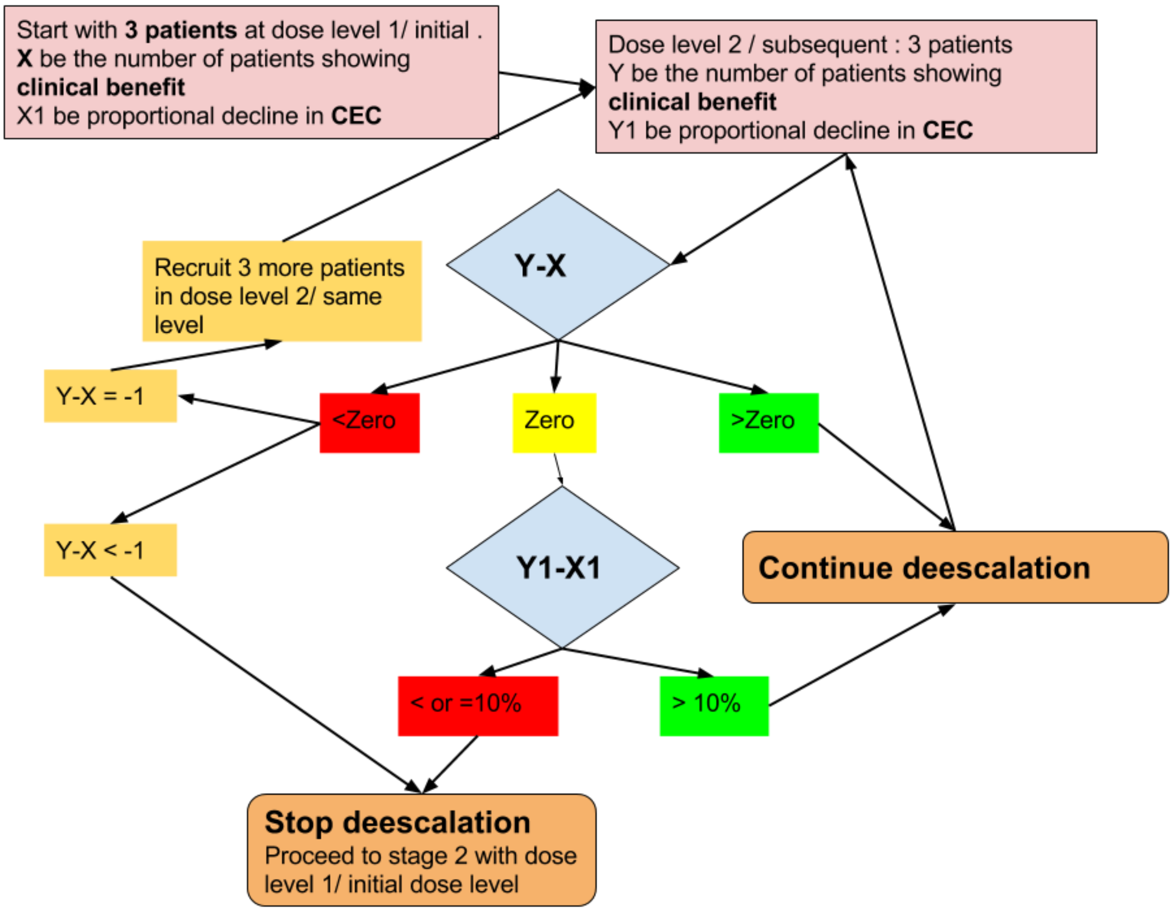

Figure 2 : Decision tree.

4.8 PK-PD (Pharmacokinetics- Pharmacodynamics) study of Methotrexate

Pharmacokinetics of methotrexate is well described by two-compartment model. There are many publications describing PK-PD model for methotrexate. one study has described relationship between methotrexate concentration and homocysteine (HCY) levels. An indirect response model was used to describe the HCY concentrations. An E_{max} model was used to link the PK and PD model (Figure 3).

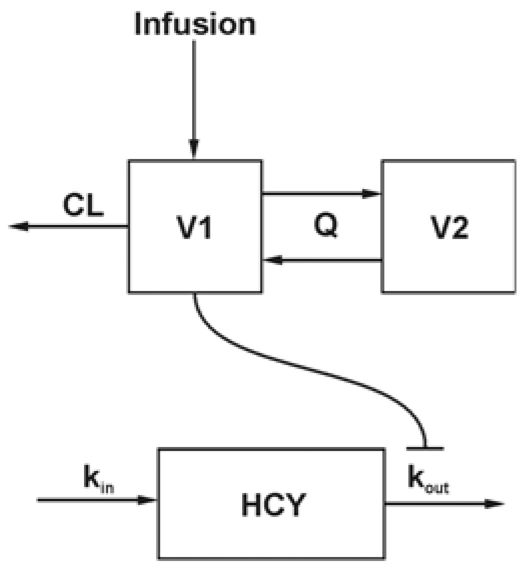

Figure 3. Scheme of the PK/PD model (CL=total body clearance; Q=intercompartmental clearance; V1=volume of the central compartment; V2=volume of the peripheral compartment; HCY=homocysteine; k_{in}=HCY formation rate constant; k_{out}=HCY elimination rate constant).

So far, no study is reported to describe PK-PD model for anti-angiogenic effect of methotrexate. Developing a model which describes relationship between methotrexate concentration and CESs and biomarkers like VEGF, sVEGFR-2 and sVEGFR-3 will be helpful to establish the OBD of methotrexate.

5 Rationale for the study

In the first stage of the study a metronomic dose of methotrexate would be selected based on both clinical and laboratory endpoints. This would be the first study which would methodically estimate the most optimal biologically effective metronomic dose of a drug.
In the second stage the clinical outcomes of this metronomic regimen would be studied. The mechanism of action, predictors of response and resistance to metronomic chemotherapy would be identified. In addition the pathways which cause resistance would be identified this would open avenue for more research and new drug combinations. Hence this research plan, would tackle an unmet need in oral cancer and would pave way for making a more efficient , accessible and affordable treatment for advanced oral cancers.

6 Hypothesis

Stage 1: Whether a lower dose of methotrexate is clinically and biologically more effective than 15 mg /m2 weekly dose? *The dose of methotrexate associated with maximum CBR at 2 months and/or decline in CEC at D7 of first cycle would be selected .*

Stage 2 : Whether MCT-E combination of celecoxib, erlotinib and methotrexate (in OBD) would lead to a median PFS of > 3 months in platinum insensitive oral cancers?

7 Objectives

7.1 Primary objective

1. Stage 1 : To find out the optimal biological and clinically effective metronomic dose (OBD) of methotrexate when given in combination with celecoxib and erlotinib
2. Stage 2 : To find out the median PFS (Progression free survival) in recurrent oral cancer patients with the combination of celecoxib, erlotinib and methotrexate in OBD

7.2 Secondary objective (both stages)

1. To find out the frequency of maximum grade of toxicity (hematological and non hematological) in accordance with CTCAE (common terminology criteria for adverse events, NCI,USA) version 4.03 with the combination metronomic therapy
2. To study the evolution in serum and tissue biomarker levels during metronomic chemotherapy and their relationship to disease control
    a. Serum angiogenic biomarkers : VEGF, VEGFR1-3, Tie-2, Angiopoietin 1-2, Thrombospondin 1, Vasoinhibin, Angiostatin, Endostatin ,IL-8, Circulating endothelial cells and Endothelial progenitor cells,CAIX,osteopontin,
    b. Tumor tissue marker : Thymidine phosphorylase, Hypoxia inducible factor I-alpha, DLL, SPARC , FOX-p3 Treg
3. To develop Pharmacokinetics-Pharmacodynamics (PK-PD) Model of methotrexate using CECs and serum angiogenic biomarkers as PD endpoints

7.3 Tertiary objective (both stages)

To study gene expression changes in the tumor tissue during MCT-E to identify responders and mechanisms of resistance.

1. To analyze HPV, markers of inflammation, cell proliferation, apoptosis and hypoxia to evaluate the association with treatment response.
2. To identify the transcriptomic alterations and to delineate the genes/gene clusters which are differentially regulated in response to the treatment.
3. To carry out global miRNA profiling in tissue and serum samples to identify miRNAs differentially regulated in response to the treatment.

8 Study Design

Non randomized open label stage I using novel de-escalation schema would be followed by non randomized open label stage II

9 Study Setting

Patients will be recruited at Tata Memorial Hospital / ACTREC which is a tertiary cancer centre located in Mumbai , India

10 Eligibility Criteria

10.1 Inclusion Criteria

1. Participants must have histologically confirmed squamous cell cancers of the oral cavity warranting palliative chemotherapy.
2. Patients should have failed within 1 month of surgery/ radiation or within 6 months of platinum based systemic therapy who couldn't afford cetuximab we offer metronomic chemotherapy at present.
3. Participants must have measurable disease, defined as at least one lesion that can be accurately measured in at least one dimension (longest diameter to be recorded for non-nodal lesions and short axis for nodal lesions) as ≥20 mm with conventional techniques or as ≥10 mm with spiral CT scan, MRI, or calipers by clinical exam.

4. Age : Any age above 18 years. No maximum age.
5. ECOG performance status ≤2
6. Life expectancy of less than 6 months with standard palliative treatments.
7. Participants must have normal organ and marrow function as defined below:
    a. Leukocytes ≥3,000/mcL
    b. Platelets ≥100,000/mcL
    c. Total bilirubin < 1.5 × institutional upper limit of normal
    d. AST(SGOT)/ALT(SGPT) ≤2.5 × institutional upper limit of normal
    e. Calculated Creatinine clearance > 30 ml/min
8. The effects of erlotinib , oral methotrexate and celecoxib on the developing human fetus are unknown. For this reason and because all these agents used in this trial are known to be independently teratogenic, women of childbearing potential and men must agree to use adequate contraception (hormonal or barrier method of birth control; abstinence) prior to study entry and for the duration of study participation. Should a woman become pregnant or suspect she is pregnant while she or her partner is participating in this study, she should inform her treating physician immediately. Men treated or enrolled on this protocol must also agree to use adequate contraception prior to the study, for the duration of study participation, and 6 months after completion of protocol.
9. Both men and women of all races and ethnic groups are eligible for this trial.
10. Ability to understand and the willingness to sign a written informed consent document

10.2 Exclusion Criteria

1. Participants who are receiving any other investigational agents.
2. Patients with history of aspiration pneumonia or who are unable to swallow tablets
3. Primary sites of malignancy in major salivary gland or nasopharynx or skin
4. Patients receiving methotrexate for other indications not limited to rheumatoid arthritis
5. Patients who had received long term Cox-2 inhibitors (more than 3 month continuous usage) will be excluded
6. Patients with hypercalcemia at presentation (defined as a corrected serum calcium of > 10.5 mg/dL).

7. Patients with QTc prolongation defined as QTc interval greater than 440 ms in males and 480 ms in females in view of risk of sudden cardiac death associated with use of celecoxib.
8. History of allergic reactions attributed to compounds of similar chemical or biologic composition to any agents used in study.
9. Uncontrolled intercurrent illness including, but not limited to, hypertension, tuberculosis, diabetes, ongoing or active infection, symptomatic congestive heart failure, unstable angina pectoris, cardiac arrhythmia, renal failure (on dialysis), active gastrointestinal bleeding, cerebrovascular accidents, inflammatory bowel disease, known hyperkalemia (CTCAE version 4.02 grade 3 or above which is persistent over 1 week) or psychiatric illness/social situations that would limit compliance with study requirements.
10. Pregnant women and breastfeeding women are excluded from this study because celecoxib / erlotinib and methotrexate are agents with the potential for teratogenic or abortifacient effects. Because there is an unknown but potential risk for adverse events in nursing infants. These potential risks may also apply to other agents used in this study.
11. HIV-positive, Hepatitis B and C seropositive patients are excluded from this study.
12. Patients with previous history of other cancers.

11 Interventions

11.1 Prerequisite for administration of metronomic therapy

Patients meeting the inclusion criteria who give written informed consent will be started on metronomic chemotherapy (MCT) with erlotinib, celecoxib and oral methotrexate. Patients on MCT would be monitored for response (RECIST version 1.1) and toxicity (CTCAE version 4.03) at C1D8, D30 and subsequently monthly till progression. Response at 2 months will considered for stage 1 study decisions on selection of dose level. In patients with intolerable side effects or progression during MCT (either clinical or radiological), the protocol drugs would be stopped and further treatment would be according to the institutional protocols .

Pretreatment Criteria

All patients will have to fulfil the following criteria prior to starting MCT. Each 4 weeks of treatment would be considered as one cycle.

Prior to first cycle:

1. Hemoglobin: > 10 g/dL
2. Total Leucocyte Count : > 3000 / cumm
3. Platelet Count : > 1,00,000 / cumm
4. AST / ALT : < 2.5 times upper normal limit of institutional value
5. Bilirubin: Total < 1.5 times upper normal limit of institutional value
6. Calculated serum creatinine clearance above 30 ml/min

Prior to subsequent cycles:

1. Hemoglobin: > 8 g/dL
2. Total Leucocyte Count : > 2000 / cumm
3. Platelet Count : > 80,000 / cum
4. AST / ALT : < 2.5 times upper normal limit of institutional value
5. Bilirubin: Total < 1.5 times upper normal limit of institutional value
6. Calculated serum creatinine clearance above 30 ml/min
7. Resolution of other toxicities to ≤ Grade I.

11.2 Metronomic chemotherapy administration

Chemotherapy will be administered with tablet erlotinib 150 mg (fixed dose) PO OD daily, capsule celecoxib 200 mg (fixed dose) PO BD daily and oral weekly methotrexate.

In stage 1 : The dose of methotrexate will be according to the dose level

In stage 2 : The dose of methotrexate will be the dose selected from stage 1

All chemotherapy medications will be taken 1 hour before breakfast. The methotrexate is available in 2.5 mg tablets. Hence the dose of methotrexate will be rounded of to the nearest lower numerical which is a multiple of 2.5. This could enable delivery of methotrexate without the need for breaking or crushing the tablets. All methotrexate tablets will be taken together.

Missing dose

In case the drugs are not taken prior to breakfast then they can be taken 2 hours after food. If the dose of erlotinib or celecoxib is missed at schedule time it can be taken within 12 hours on the schedule day. However the gap between doses taken on two consecutive days should be more than 12 hours. If the dose of erlotinib or celecoxib is missed on any day then it won't be compensated. If the patient cannot take for any reason the schedule medications (erlotinib and celecoxib) for more than 14 days then he would be withdrawn from the protocol.

In case of oral methotrexate as it would be given weekly if the the patient misses the schedule time of 1 hour before breakfast it can be taken at any time during the schedule day. If the scheduled day is miss it can be taken administered within next 2 days.

Delivery setting

The cycles may be delivered in inpatient and/or outpatient basis. Reported adverse events, potential risks and appropriate dose modifications are described in Section 13. No investigational or commercial agents or therapies other than those described below may be administered with the intent to treat the participant's malignancy.

The starting dose of methotrexate will be 15 mg/m2. The dose of methotrexate in a given patient will remain fixed in the subsequent cycles subject to the dose modification criteria mentioned below. Dose de escalation will be done for methotrexate only as per the schedule mentioned below. Once the clinically effective and biologically optimal dose of methotrexate is discovered then this dose will be administered in subsequent stage 2 study.

Dose Level	Dose of Methotrexate*
Level 0 – Starting Dose	15 mg/m^2
Intermediate dose level between dose level 0 & 1 (if required)	13 mg/m2
Level 1	12 mg/m^2
Intermediate dose level between dose level 1 & 2 (if required)	10 mg/m2

Level 2	09 mg/m^2
Intermediate dose level between dose level 2 & 3 (if required)	07 mg/m2
Level 3	06 mg/m^2
Intermediate dose level between dose level 3 & 4 (if required)	04 mg/m2
Level 4	03 mg/m^2

Table 2: Dose levels of methotrexate for dose de escalation.

Day	Agent	Dose	Route	Schedule
D1	Tablet Erlotinib	150 mg OD	PO 1 hour prior to breakfast	D1- D28
	Capsule Celecoxib	200 mg BD	PO 1 hour prior to breakfast	D1- D28
	Tablet Methotrexate	Stage 1 : Dependent of dose level , OD<br>Stage 2 : The dose of methotrexate selected from stage 1	PO 1 hour prior to breakfast	D1,D8,D15,D22

Table 3: Chemotherapy schedule. Schedule will be repeated at 4 weekly intervals subject to resolution of toxicities and with dose modification mentioned below.

General Concomitant Medications and Supportive Care Guidelines

1. Loperamide for patients with Grade II or more diarrhoea following chemotherapy.
2. Doxycycline for patients with Grade II or more rash following chemotherapy
3. Topical steroids for patients with Grade II or more rash following chemotherapy
4. Oral steroid for patients with Grade II or more nausea or vomiting following weekly methotrexate

5. Antibiotics can be administered to patients presenting with febrile neutropenia as per the institute antibiotic policy.
6. Patients should continue pre-existing medications for other comorbid conditions. However the same should be noted for any significant interactions.

Duration of Therapy

Patients who have radiological progression but achieve symptomatic relief and insist on continuation of MCT may be continued the same on compassionate ground.

Followup

Patients will receive MCT at 4 weekly intervals continuously till progression or intolerable side

As this is a stage I/II trial hence further followup after treatment progression will be conducted as per institutional norms.

11.3 Removal of Patients from Protocol

Patients will be removed from Protocol treatment in the event of:

1. Inter-current illness that prevents further administration of treatment,
2. Participant unable to start any MCT cycle within 14 days of last cycle.
3. Toxicity which leads to discontinuation of one or more chemotherapy agents
4. Participant demonstrates an inability or unwillingness to comply with the oral medication regimen and/or documentation requirements
5. Participant decides to withdraw from the study
6. General or specific changes in the participant's condition render the participant unacceptable for further treatment in the opinion of the treating investigator.
7. Logistical constraints prevent from delivering chemotherapy e.g unavailability of chemotherapy medications or allergy to it.

The reason for study removal and the date the participant was removed must be documented in the study-specific case report form (CRF). Alternative care options will be discussed with the participant. In the event of unusual or life-threatening complications, participating investigators must immediately notify the Principal Investigator (or Protocol Chair)

In general, treatment interruptions should be avoided by preventive medical measures and nutritional, psychological, and emotional counseling. Treatment breaks, including indications, must be clearly documented on the treatment record.

11.4 Toxicities and Dosing Delays / Dose Modifications

All patients will be evaluable for adverse events due to chemotherapy during the study. The schedule for monitoring of adverse events is mentioned under the time and events table. All toxicities will be evaluated as per the NCI CTCAE 4.03 criteria. The final dose modification according to the following tables should be based upon the worst grade of adverse event experienced. If any of the treatment agents is held or discontinued for toxicity or intolerance, further protocol directed treatment will be stopped. Held doses of of any agent will not be made up at a later date. If more than one dose reduction applies, then use the most stringent (i.e., the greatest dose reduction). Doses that are reduced at any point during a cycle will not be re-escalated. Grade I toxicities will not result in dose modifications. Patients will be removed from the protocol in the event of discontinuation of any of the agents mentioned in the protocol as per criteria mentioned below.

Hematological Toxicities

Hematological toxicity will be monitored in accordance with following time points
Cycle 1: Complete hemogram for monitoring of toxicity will be done on D8 (D7-D10)
Subsequent Cycles : Complete hemogram for monitoring of toxicity will be done at start of each cycle.
Since hematological toxicities is caused by methotrexate , dose modifications should be done for both agents according to dose levels defined in Table 6 for the most severe haematological toxicity.
The doses used for subsequent cycles in case of hematological toxicity are

	Methotrexate	Celecoxib	Erlotinib
ANC > 1 (X109/L) and Platelet > 50,000	100% dose	100% dose	100% dose
ANC < 1 (X109/L) and/or Platelet < 50,000	75% dose	100% dose	100% dose

Table 4: Table showing dose modifications with hematological toxicities experienced.

Non Hematological Toxicity

MCT has very limited non hematological side effects.The subsequent subsections shows the dose modifications that should be done for derangements of metabolic parameters and non hematological toxicity during the treatment.

Nausea & Vomiting

Nausea and vomiting are unlikely. Primary prophylaxis against nausea and vomiting is not required as per the schedule mentioned in Treatment schedule above. Patients with Grade III – IV nausea and vomiting should have the dose withheld till the nausea, vomiting subsides to Grade I or less. Patients who have had one episode of Grade III / IV nausea or vomiting should have primary prophylaxis with addition of ondansetron (4 mg BD) from the subsequent cycles of methotrexate chemotherapy. If patients continue to have Grade III- IV vomiting despite instituting antiemetic ondansetron then from the subsequent cycles of methotrexate chemotherapy additive antiemetics can be added. If inspite of high risk antiemetic prophylaxis patients continue to have vomiting (Grade III-IV) then methotrexate should be discontinued.

Nephrotoxicity

Nephrotoxicity will be monitored in accordance with following time points

Cycle 1: Renal function test (inclusive of serum urea, creatinine ,Na and K) for monitoring of toxicity will be done on D8 (D7-D10)

Subsequent Cycles : Renal function test (inclusive of serum urea, creatinine ,Na and K) for monitoring of toxicity will be done at start of each cycle.

The dose reduction in case of nephrotoxicity will be done in accordance with below mentioned table.

Serum creatinine clearance	Methotrexate	Celecoxib	Erlotinib
> 30 ml/min	100 % dose	100 % dose	No dose adjustment
15-30 ml/min	50% dose	Avoid	No dose adjustment
< 15 ml/min	Avoid	Avoid	No dose adjustment

Table 5: Dose reductions in case of nephrotoxicity

Hepatic Dysfunction

Patients should be monitored for liver dysfunction prior to each chemotherapy cycle. Dose modifications will be made as per the following table.

Bilirubin		ALT/AST	Methotrexate	Celecoxib	Erlotinib
2-4 x ULN	And/or	>4 x ULN	75% dose	50 % dose	50 % dose
>4 x ULN	And/or	>4 x ULN	Avoid	Avoid	Avoid

Table 6: Dose adjustments to be made as per hepatic function.

The above biochemical parameters except Creatinine Clearance will be monitored prior to each cycle. Patients will have creatinine clearance measured prior to each cycle in case of any episode of Grade II renal toxicity prior to each cycle of chemotherapy.

Potential Drug Interactions

Medscape interaction checker calculator application would be used for checking potential drug interactions each visit . The drugs having significant interactions would be substituted unless the risk of using the medicine concurrently exceeds the benefit.

11.5 Compliance Criteria

Treatment Duration for the first and second cycle of MCT will be considered for compliance in stage I. As the primary objective is to evaluate the disease control after the second cycle hence treatment duration on the subsequent cycles are not considered, although every effort will be made to ensure that all cycles are delivered in a timely fashion.

Per Protocol: Treatment of first 2 cycle should be completed within 8 weeks. In case of delay of any nature except due to toxicity or progression of disease if the treatment is completed within 9 weeks (with all 8 weekly doses of methotrexate administered and not more than 6 cumulative doses of erlotinib and celecoxib missed) the patients would be considered compliant

The compliance with oral treatment will be assured by verbal confirmation from the patient and his closest caretaker .

11.6 Imaging Plan

Patients will undergo diagnostic CECT neck and thorax prior to inclusion in the study to rule out distant metastases. Use of other imaging modalities including bone scan / PET CT for detection of metastases is left to the discretion of the treating physician based on the clinical findings for a given patient. At 2 monthly intervals the patient would undergo radiological response assessment imaging if the patient has not grossly clinically progressed.

12 Study procedure

12.1 Clinical Intervention

Patients on MCT would be monitored for response (RECIST version 1.1) and toxicity (CTCAE version 4.03) at C1D8, D30 and subsequently monthly till progression.

12.2 Laboratory interventions

Sample collection:

1. **Tumor tissues will be collected at baseline (+1 month prior to 2 days post MCT start), C1D8 (+/- 2 days) and at progression.** Fresh(~20-30 mg) and formalin fixed tumor tissues will be used. Tumor content will be assayed and tissues having less than 60% of tumor will not be used for microarray analysis. Fresh tumor tissue will be subjected to extraction of DNA and total RNA while fixed tumor tissues will be used for IHC.

2. **20 ml of blood will be collected (5 ml in 3 EDTA tube and 5 ml in plain tube) at baseline collected at baseline (+7 days prior to 1 days post MCT start) , C1D8(+/- 2 days) and at progression.** 1 EDTA tube each will be used for Flow cytometry, Microarray and ELISA. Fourth tube will be a reserve. 10 ml of blood will be used for separation of plasma (5 ml) and serum (5ml) which will be stored at -80^{0}C, used for analysis of various parameters.
    a. PK-PD Sample size collection restricted to stage 1
        i. 5 ml blood will be collected at each time point. Immediately after collection, blood samples will be kept on ice till they are further process to separate plasma by centrifugation at 4^0 C. Plasma samples will be stored

at -80^{0}C till further analysis of drug levels (Bioanalysis using LC-MS/MS) and levels of CECs and biomarkers.

DRUG	TIME POINTS FOR SAMPLING	No. of Draws	Cycle and Day
Methotrexate	0, 0.5, 1, 1.5 , 4, 6, 8, 24, 30 hr	9	C1D1,

3. **At the time of each monthly D30 (+/- 7 days) follow up 10 ml of blood will be collected in EDTA tube for the first 3 months for Flow cytometry and ELISA .**

Flow cytometry procedure

Timeline for doing flow cytometry

All patients would undergo serum CEC/EPC at least 4 day before administration of oral therapy, then at at D8 (+/- 2 days) , D30 (+/- 5 days), 30 days interval for first 3 months (+/- 7 days) and at progression.

Procedure

Collection of blood sample : 5 ml of blood sample from peripheral vein in EDTA vacutainer (first 2-3 ml would be discarded)

Processing of sample

The blood would be processed within 24 hours of collection preferably within 4 hours

Dilution of blood : If TLC count is more than 20,000 per microliter than dilution would be used. If not than then the whole blood with dilution would be subjected to further steps

Blocking serum (to inhibit nonspecific antibodies) : Fc receptor blocking agent incubation for 30 min (The amount and time would vary according to the kit)

Incubation with monoclonal antibodies tagged with fluorescence : 4 degree , 30 min in a dark place (The amount and time would vary according to the kit)

1. CD 146 (endothelial cell marker, marker also seen on T cells)
2. CD 45 (Antigen present on T cell but not on endothelial cells)
3. CD 34 (endothelial and stem cell marker)
4. CD 31 (Endothelial cell marker but absent in stem cell)

5. CD 133 (progenitor cell marker)
6. Viability stain (7 AAD) (apoptosis marker)

Wash and red blood cell lysis : Add 2 ml of NH4CL freshly prepared. (1X NH4CL=1800 microliter D.water + 200 microliter 10 X NH4CL)

Run in Flow cytometer : Instrument for Flow cytometry; 4-6 colour Flow cytometer, FACSCalibur (Becton & Dickinson, USA)

Interpretation of results

1. 1 million events would be recorded
2. Debris and nonviable cell will be excluded by gating in FSC and SSC plot for 7 AAD
3. Gates would be drawn so that CD 146 + and CD 45 (-/dim) would be selected for further analysis
4. This gated population would be further subjected to gating for selection of CD 34 (+), CD 31 (+) cells
5. These cells would than be gated for CD 133
6. The population of CECs and EPC will be identified as per below
    a. CECs : CD 146 (+), CD 45 (-/dim), CD 34 (+), CD 31 (+) and CD 133 (-)
    b. Endothelial progenitor cells : CD 146 (+), CD 45 (-/dim), CD 34 (+), CD 31 (+) and CD 133 (+)
    c. Cut off value for CD31 (+) would be kept at 4*10‘1 and at 1*10‘1 for 7AAD (+); CD146(+) and CD133 (+)

Absolute number would be calculated by following formula = (Percentage of positive cells on total events) / (TLC count)

ELISA

Procedure

Collection of blood sample : 5 ml of blood sample from peripheral vein in EDTA vacutainer

Processing of sample

Serum extracted from 5 ml of EDTA blood sample would be processed together at the end of clinical study protocol. Hence these serum samples would be stored at -40 degree celsius.

Sample will be processed as per the individual antibody ELISA kit manufacturers guideline on luminex platform.

Tissue based markers (IHC or microarray)

Analysis of markers of hypoxia and angiogenic markers in tumor tissues by IHC

Positive cells per different fields will be counted and graded and the results will be reconfirmed by two pathologists. Hypoxia status will be evaluated by using antibodies against HIF1α and its downstream targets carbonic anhydrase IX and VEGF. In addition IHC would be done for SPARC, Thymidine phosphorylase and DLL-4 (Table 1). Human kidney tumor or human placenta will be used as positive controls for the immunohistochemical analysis of HIF1α.

Detection of HPV

Over-expression of $p16^{INK4a}$ is a surrogate marker for HPV detection hence the samples will be screened by using antibody (CINtec histology $p16^{INK4a}$ kit) as described by Smeets et al . Positive $p16^{INK4a}$ expression is defined as strong and diffuse nuclear and cytoplasmic staining in 70% or more of the tumor cells. In the cases of positive p16 expression, the presence of HPV genomic DNA in oral tumour samples will be detected by Nested PCR using general primer pair MY09/11 and GP5+/GP6+. DNA from human cervical carcinoma *cell line*s SiHa, CaSki (HPV-16 containing cell line), HeLa (HPV-18 containing cell line) will be used as a positive control. House keeping gene beta globin gene will be used as an internal control to test the amplifiability of the template DNA. HPV positive cervical cancer tissues will be used as a positive control when the tissues will be analyzed by *immunohistochemistry* (IHC). All PCR positive samples will be further analyzed for the presence of transcriptionally active HPV by RNA SCOPE assay.

Cell proliferation markers by IHC

Ki67 is the nuclear protein and a transcription factor associated with cellular proliferation while Cyclin D1 regulates cyclin dependent kinases which are involved in the regulation of different cell proliferation pathways. Hence these markers will be studied

Apoptosis markers by IHC

Members of Bcl2 family of proteins, whose members have either proapoptotic (Bax) or antiapoptotic (Bcl2) function, act in part by governing mitochondrial cell death through cytochrome C release. Hence ratio of these proteins will be used to measure the apoptotic index and will be calculated as ratio of no. of Bax positive cell / no. of Bcl2 positive cells.

Inflammation markers by IHC

Cyclooxygenase 2 (COX2) is the key enzyme in prostaglandin biosynthesis, and is responsible for the inflammation.

Microarray based Gene Expression Analysis

Global gene expression profiling will be carried out on the tissue samples. RNA will be isolated from the tumor tissue samples using the QIAGEN RNeasy Mini kit according to the instructions of the manufacturer. RNA with RIN value more than 7 will be used for analysis. Gene expression arrays from Agilent Technologies (4x44k) will be used for proposed analysis. The comparison will be carried out analyze the differentially expressed genes before starting the therapy and at the end of the therapy. Results of microarray data will be validated by TaqMan quantitative real-time PCR and immunohistochemistry .

Microarray based global miRNA profiling of serum.

Total RNA will be extracted from 400µl of serum from patients using a mirVana PARIS kit (Ambion) and finally eluted into 100 µl of preheated (95°C) elution solution according to the manufacturer's protocol. The miRNA microarrays manufactured by Agilent Technologies will be used. There are a total of 1205 human and 144 human viral miRNAs on the Agilent Human miRNA Microarray. Total of 100 ng RNA will be labelled and hybridised using the Human microRNA Microarray Kit protocol. Differentially expressed miRNAs will be validated by TaqMan quantitative real-time PCR (TaqMan MicroRNA Assays; Applied Biosystems).

Need for multiple biopsies

This genomic analysis will help us to understand the mechanism of action, pathways involved in response and resistance. Three biopsies are required. The reason for these biopsies are

1. The baseline biopsy will help us understand about gene expression in platinum insensitive oral cancers, which genes are upregulated and which are down regulated. This information can not help us in understanding mechanism of action. As the drug exposure has not yet happened. It will gives the baseline state of gene expression in platinum insensitive oral cancers.
2. The biopsy on next visit that is 7th day will then be taken and a gene expression analysis will be done. This gene expression analysis will be compared with the baseline gene expression analysis. This would tell us that after exposure to this combination of MCT_E which genes are upregulated and which are down regulated. This information will then be used to derive pathways which are upregulated or downregulated post exposure to metronomic chemotherapy. So base on these two biopsies we would get the pathways which are activated or deactivated by MCT ie mechanism of action.

   a. The day 7 biopsy is selected as metronomic molecular actions are genetically expressed by day 6. [51, 52]

3. Now that we would know which pathways are helpful in metronomic action. We would do the third biopsy at progression. The comparison of gene expression of third biopsy would be done with the second one. This will tell us which pathways are responsible for resistance

13 Time and Events Table

All baseline investigations should be performed prior to the start of the chemotherapy. Hematological and biochemical investigations should be obtained within one week of start of chemotherapy. Imaging investigations may be performed within 2 weeks of the start of chemotherapy. All investigations indicated on the chart below will be repeated on the day of the chemotherapy prior to the administration of the chemotherapy.

Day	Pre-study	C1D1	C1D8	D1 of first 3 cycles	D1 of subsequent cycles	Progression
Eligibility Screen	✔	-	-	-	-	-
Informed Consent	✔	-	-	-	-	-
Multi speciality Board	✔	-	-	-	-	-
History	✔	-	✔	✔	✔	✔
Physical Examination	✔	-	✔	✔	✔	✔
Toxicity Charting	-	-	✔	✔	✔	✔
Complete Hemogram	✔	-	✔	✔	✔	✔
Renal Function	✔	-	✔	✔	✔	✔
Liver Function	✔	-	✔	✔	✔	✔
Serum Electrolytes (Ca2+ / Mg2+)	✔	-	✔	-	-	-
Biopsy	✔	-	✔	-	-	✔
Serum markers (ELISA +	✔	-	✔	✔	-	✔

Flow)						
MRI/CT Head Neck	✔	-	-	✔	✔	✔
MCT Chemotherapy	-	✔	✔	✔	✔	-

Table 7 : Study calendar

14 Study timeline

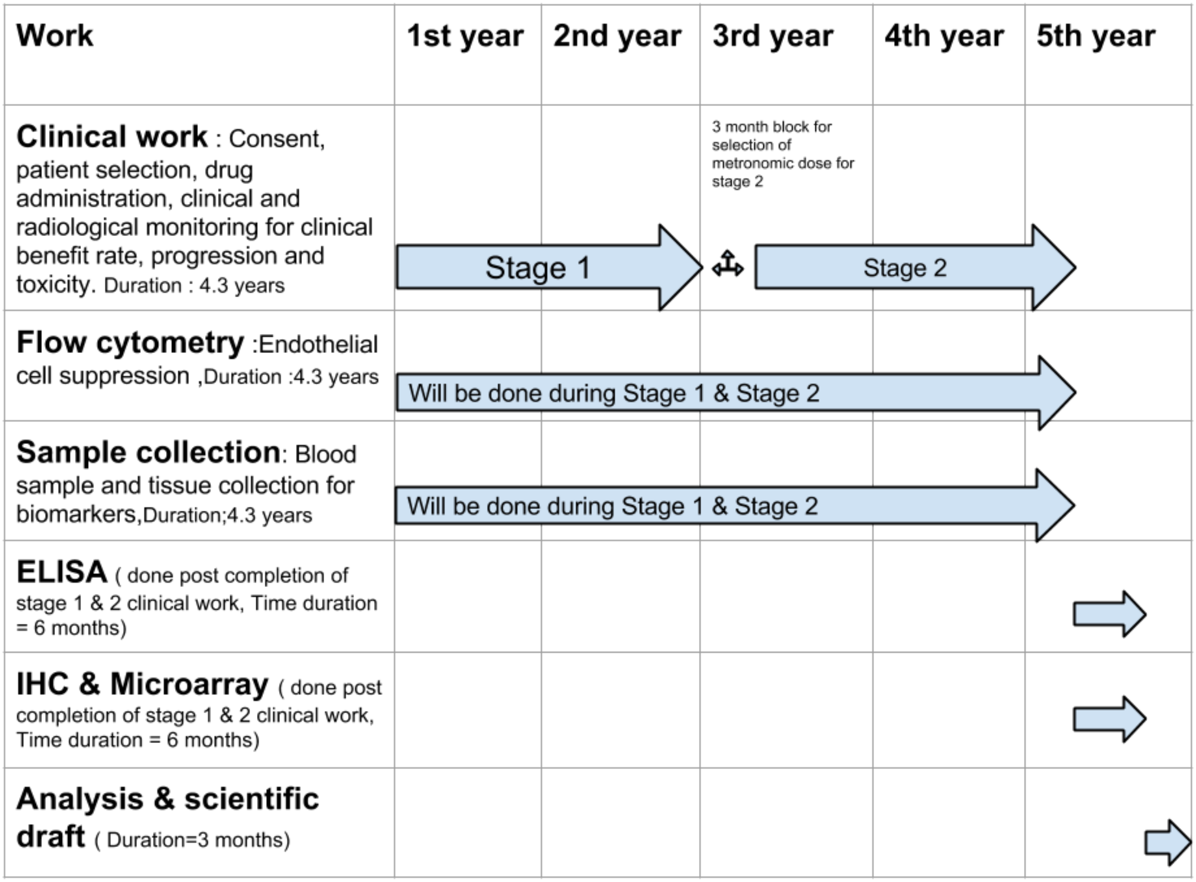

15 Sample size calculation

Total sample size : Stage 1 (20-30 patients) + Stage 2 (76 patients). In stage the continuous reassessment model will be used hence the sample size would be maximum of 30 and minimum around 20. The exact calculations done in both stages are described below in details.

Stage 1 : Flexible-finite sample based approach will be adopted to select the circulating endothelial cells (CEC) interval boundaries and further to use optimum interval design. During the trial, we will continuously update the posterior estimates of decline in CEC and clinical benefit rate and assign patients to the most appropriate dose combination (Figure 1). The Bayesian adaptive screening design will be used to simultaneously select among possible treatment dose combinations. The design is based on formulating the selection procedure as a Bayesian hypothesis testing problem in which the superiority of each treatment combination is equated to a single hypothesis. During the trial conduct, we intend to use the current values of the posterior probabilities of all hypotheses to adaptively allocate patients to treatment combinations. The dose-finding decision will be decided on the twin endpoints the clinical benefit rate at 2 months and proportional decline in CEC at D8 of first cycle.[53] Figure 2 will be used for decision making.

Stage 2: It was assumed that the 3-month progression-free survival (PFS) rate of a standard cancer therapy is 0.50 (H0). An improvement to 0.60 would be considered clinically significant (H1). Assume both the null and alternative PFS distributions follow an exponential distribution. The 1-sided type I error rate of 0.05 and type II error rate of 0.1. Prior assumption includes a fixed enrollment rate of 3 patients per month. We require a stop of 3 months at the end of Stage 1. The library(“OptInterim”) has been used to calculate the sample size. A 20%extra patient would be taken to account for lost to follow up data. Hence **a total of 76 (63 patients +13 patient) sample size is found suitable to carry out the study for stage II with single arm drug therapy.**

16 Outcome measures

16.1 Primary outcome :

In stage I the OBD of methotrexate would be identified using response assessment criteria RECIST version 1.1 and flow cytometry measurement of CEC. So a twin primary endpoints of clinical benefit rate at 2 months (CR or PR or SD persisting till 2 months) and proportional decline in CEC will be used for determination of OBD.

In stage 2 the PFS would be measured in days from date of recruitment in the study till the date of progression (clinical or radiological or both). Median PFS would be estimated by Kaplan Meier survival method.

16.2 Secondary and tertiary outcomes:

Toxicity would be assessed at clinical visits . Relation between biomarkers and disease control (clinical benefit rate and progression free survival) would be explored by using a suitable statistical test selected on the basis of type of data (continuous, ordinal) and its distribution. Multiplicity bonferroni correction would be used.

17 Data Collection Methods

Patient eligibility for the trial will be documented in a separate eligibility checklist (Appendix). Each trial participant will be assigned a study specific identification which will be used for referring to that patient in the study duration subsequently. A study specific CRF (Appendix) will be used for these patients to capture the baseline clinical and laboratory parameters. Patients will be assessed on day D8 of the 1st cycle of chemotherapy using a custom designed proforma (Appendix) which will capture the toxicity encountered in the patient in the last7 days. After the first cycle another proforma (Appendix) will be used to capture toxicity in the last chemotherapy cycle.Other procedures and tests done for this protocol including imaging will be detailed in the existing departmental forms. A copy of these forms will be kept along with the patient record for the trial. Results of hematological and biochemical tests will be recorded in the toxicity recording proformas mentioned above.

18 Data Management

The trial specific CRF and other forms will be kept in a secure almirah in the Department of Medical Oncology. Data entry will be done on a trial specific electronic database maintained on the hospital local area network. Double data entry will be done by two trial coordinators. Data management will be done in accordance with the hospital data safety and monitoring board guidelines.

19 Quality assurance

Chemotherapy Quality Assurance

The standards proposed by ASCO/ONS Chemotherapy Safety Standards for Drug preparation and administration will be followed. At least two medical personnels will verify the patient identification, drug names, dose, route of administration and expiration dates/times and appearance and physical integrity of the drug to be administered prior to each cycle. They will also sign the chemotherapy delivery chart independently to verify the above.

Toxicity Assessment

All toxicities will be graded as per the CTCAE v 4.03 criteria. Disagreement between grading by two observers in the toxicity levels will be resolved through discussion with a reason behind disagreement noted for each event and the resolution adopted. In order to ensure complete documentation of toxicity a prespecified toxicity CRF will be used and recorded for each patient.

20 Monitoring

20.1 Data Monitoring

The institutional data monitoring and safety board (DSMB) will be responsible for oversight of the data. A trial specific data monitoring committee may be formed at the discretion of the DSMB to monitor the trial progress.

20.2 Interim Analysis

As this is a stage I/II study , interim analysis will be done at after completion of stage 1.

21 Harms

An adverse event will be defined as any untoward medical occurrence in a subject without regard to the possibility of a causal relationship. Adverse events will be collected after the subject has provided consent and enrolled in the study. If a subject experiences an adverse event after the informed consent document is signed (entry) but the subject has not started to receive study intervention, the event will be reported as not related to study drug. All adverse events occurring after entry into the study and until hospital discharge will be recorded. An adverse event that meets the criteria for a serious adverse event (SAE) between study enrollment and hospital discharge will be reported to the local IRB [institutional review board] as an SAE.

If chemotherapy is discontinued as a result of SAE, study personnel will document the circumstances and data leading to discontinuation of treatment. A serious adverse event for this study is any untoward medical occurrence that is believed by the investigators to be causally related to study-drug and results in any of the following: Life-threatening condition (that is, immediate risk of death); severe or permanent disability, prolonged hospitalization, or a significant hazard as determined by the data safety monitoring board. Serious adverse events occurring after a subject is discontinued from the study will NOT be reported unless the investigators feels that the event may have been caused by the study drug or a protocol procedure. Investigators will determine relatedness of an event to study drug based on a temporal relationship to the study drug, as well as whether the event is unexpected or unexplained given the subject's clinical course, previous medical conditions, and concomitant medications.

The study will monitor the following adverse events during chemotherapy cycles:

Haematological

Anemia

Leucopenia

Thrombocytopenia

Febrile neutropenia

Non hematological

Nausea

Vomiting

Oral mucositis

Pain

Xerostomia

Dysphagia

Dyspnea

Anorexia

Diarrhea

Constipation

Dysgeusia

Fatigue

Peripheral Sensory neuropathy

Peripheral Motor Neuropathy

Nail infection

Weight loss

Myalgia

Joint Pain

Alopecia

Biochemical

Increase in serum creatinine

Increase in Alanine Aminotransferase (SGPT)

Increase in Alkaline Phosphatase (ALP)

Increase in Bilirubin

Decrease in serum albumin (Hypoalbuminemia)

Increase or decrease in Serum electrolytes

Sodium

Potassium

Corrected Calcium

Magnesium

22 Auditing

The trial data will be audited at 6 monthly intervals to monitor the participant enrolment, consent, eligibility, and allocation to study groups; adherence to trial interventions and policies to protect participants, including reporting of harms; and completeness, accuracy, and timeliness of data collection. An independent auditing body will be tasked with this proposed which has been identified by the data safety and monitoring body (DSMB).

23 Ethics and Dissemination

23.1 Ethics Approval

This protocol and the template informed consent forms contained in Appendix II will be reviewed and approved by the institutional IRB with respect to scientific content and compliance with applicable research and human subjects regulations. The protocol, site-specific informed consent forms (local language and English versions), participant education and recruitment materials, and other requested documents—and any subsequent modifications — also will be reviewed and approved by the IRB. Subsequent to initial review and approval, the IRB will

review the protocol at least annually. The Investigator will make safety and progress reports to the IRB at six monthly intervals and within three months of study termination or completion. These reports will include the total number of participants enrolled and summaries of each DSMB [data safety and monitoring board] review of safety and/or efficacy.

23.2 Protocol amendments

Any modifications to the protocol which may impact on the conduct of the study, potential benefit of the patient or may affect patient safety, including changes of study objectives, study design, patient population, sample sizes, study procedures, or significant administrative aspects will require a formal amendment to the protocol. Any and all such amendments will be communicated to the institutional IRB for review and approval. Administrative changes of the protocol are minor corrections and/or clarifications that have no effect on the way the study is to be conducted. These may be communicated to the IRB at the investigators' discretion.

23.3 Consent / Assent

Patients will be given the patient information sheet by the trial investigators / nurses. The purpose and reasons behind the study will be communicated to the patient. All patients will be provided with a copy of the written informed consent as well as the patient information sheet. Consent will be recorded on video/paper as per institutional IRB guidelines.

23.4 Confidentiality

All study-related information will be stored securely at the study site. All participant information will be stored in locked file cabinets in areas with limited access. All laboratory specimens, reports, data collection, process, and administrative forms will be identified by a coded ID [identification] number only to maintain participant confidentiality. All records that contain names or other personal identifiers, such as locator forms and informed consent forms, will be stored separately from study records identified by code number. All local databases will be secured with password-protected access systems. Forms, lists, log books, appointment books, and any other listings that link participant ID numbers to other identifying information will be stored in a separate, locked file in an area with limited access.

23.5 Access to Data

The Data Management Coordinating Center will oversee the intra-study data sharing process, with input from the Data Management Subcommittee. All Principal Investigators will be given

access to the cleaned data sets. Project data sets will be housed on the project specific database created for the study, and all data sets will be password protected.

23.6 Ancillary and post trial care

Patients will be treated with supportive care and palliative care. In addition in the event of intolerable side effects or disease progression palliative radiotherapy may be used for their treatment. These costs will not be reimbursed for the patient as they are standard of care for such patients.. Patients will be treated for trial related adverse events in the hospital. Trial related compensation for death / SAEs will be provided as per institutional guidelines.

24 Dissemination Policy

The trial results will be submitted for publication in international peer reviewed journals and presented at national/international conferences. No publication restrictions will be imposed by trial sponsors. All co investigators will be given authorship on the manuscript as well as on any abstract that are reported in national/international conferences. The principal investigators will take the roles of the corresponding author and first author positions as decided mutually. Authorship criteria for other publications stemming from the study will be decided as per the relative contributions and existing institutional guidelines. The contribution of all investigators will be acknowledged in such manuscripts if they are not eligible for authorship. In addition the grant giving organizations will also be acknowledged in all publications.

25 Feasibility

On an average in TMC, 30 chemoresistant patients receive palliative chemotherapy for advanced oral cavity cancers per month. The maximum required sample size for the present study is 30 patients (6 x 5 dose levels). Assuming that only 10% of the eligible patients are willing to participate in this trial we expect 30 patients to be recruited within a period of 24 months from the date of start of recruitment. Hence around 70 patients (cumulative number required for stage I/II) would be recruited within 4 years.

26 Funding

We will be seeking intramural funding from Tata memorial Centre (TMC) as well as extramural grants from agencies like, ICMR and Wellcome Trust.

27 Risks

Side effects associated with the delivery of MCT are to be expected as risks. Previous data from India has shown that this regimen is well tolerated. We anticipate that the risk of local mucositis , loose motions and skin rash side effects may be increased with the use of erlotinib with methotrexate and celecoxib. However risk of other side effects like hematological, neurological and renal side effects are unlikely to be increased.

28 References

1. Wittes RE, Cvitkovic E, Shah J, et al: CIS-Dichlorodiammineplatinum(II) in the treatment of epidermoid carcinoma of the head and neck. Cancer Treat Rep 61:359–366, 1977

2. Campbell JB, Dorman EB, McCormick M, et al: A randomized phase III trial of cisplatinum, methotrexate, cisplatinum + methotrexate, and cisplatinum + 5-fluoro-uracil in end-stage head and neck cancer. Acta Otolaryngol 103:519–528, 1987

3. A phase III randomised trial of cistplatinum, methotrextate, cisplatinum + methotrexate and cisplatinum + 5-FU in end stage squamous carcinoma of the head and neck. Liverpool Head and Neck Oncology Group. Br J Cancer 61:311–315, 1990

4. Vermorken JB, Mesia R, Rivera F, et al: Platinum-based chemotherapy plus cetuximab in head and neck cancer. N Engl J Med 359:1116–1127, 2008

5. Rath GK, Parikh PM, Hukku S, et al: Indian Council of Medical Research consensus document for the management of buccal mucosa cancer. Indian J Med Paediatr Oncol 35:136–139, 2014

6. Pfister DG, Ang K-K, Brizel DM, et al: Head and neck cancers, version 2.2013. Featured updates to the NCCN guidelines. J Natl Compr Canc Netw 11:917–923, 2013

7. Grégoire V, Lefebvre J-L, Licitra L, et al: Squamous cell carcinoma of the head and neck: EHNS–ESMO–ESTRO Clinical Practice Guidelines for diagnosis, treatment and follow-up. Ann Oncol 21:v184–v186, 2010

8. Vermorken JB, Herbst RS, Leon X, et al: Overview of the efficacy of cetuximab in recurrent and/or metastatic squamous cell carcinoma of the head and neck in patients who previously failed platinum-based therapies. Cancer 112:2710–2719, 2008

9. Sosa AE, Grau JJ, Feliz L, et al: Outcome of patients treated with palliative weekly paclitaxel plus cetuximab in recurrent head and neck cancer after failure of platinum-based therapy. Eur Arch Otorhinolaryngol 271:373–378, 2014

10. Ignacio DN, Griffin JJ, Daniel MG, Serlemitsos-Day MT, Lombardo FA, Alleyne TA.: An evaluation of treatment strategies for head and neck cancer in a... - PubMed - NCBI. West Indian Med J 62:504–509, 2013

11. André N, Banavali S, Snihur Y, et al: Has the time come for metronomics in low-income and middle-income countries? Lancet Oncol 14:e239–48, 2013

12. Patil VM, Noronha V, Joshi A, et al: A prospective randomized phase II study comparing metronomic chemotherapy with chemotherapy (single agent cisplatin), in patients with metastatic, relapsed or inoperable squamous cell carcinoma of head and neck [Internet]. Oral Oncol , 2015Available from: http://dx.doi.org/10.1016/j.oraloncology.2014.12.002

13. Zimmermann M, Zouhair A, Azria D, et al: The epidermal growth factor receptor (EGFR) in

head and neck cancer: its role and treatment implications. Radiat Oncol 1:11, 2006

14. Thomas F, Rochaix P, Benlyazid A, et al: Pilot study of neoadjuvant treatment with erlotinib in nonmetastatic head and neck squamous cell carcinoma. Clin Cancer Res 13:7086–7092, 2007

15. Viloria-Petit A, Crombet T, Jothy S, et al: Acquired resistance to the antitumor effect of epidermal growth factor receptor-blocking antibodies in vivo: a role for altered tumor angiogenesis. Cancer Res 61:5090–5101, 2001

16. Shi S, Klotz U: Clinical use and pharmacological properties of selective COX-2 inhibitors. Eur J Clin Pharmacol 64:233–252, 2008

17. Saba NF, Hurwitz SJ, Kono SA, et al: Chemoprevention of head and neck cancer with celecoxib and erlotinib: results of a phase ib and pharmacokinetic study. Cancer Prev Res 7:283–291, 2014

18. Shin DM, Zhang H, Saba NF, et al: Chemoprevention of Head and Neck Cancer by Simultaneous Blocking of Epidermal Growth Factor Receptor and Cyclooxygenase-2 Signaling Pathways: Preclinical and Clinical Studies. Clin Cancer Res 19:1244–1256, 2013

19. Gross ND, Bauman JE, Gooding WE, et al: Erlotinib, Erlotinib–Sulindac versus Placebo: A Randomized, Double-Blind, Placebo-Controlled Window Trial in Operable Head and Neck Cancer. Clin Cancer Res 20:3289–3298, 2014

20. Kao J, Genden EM, Chen C-T, et al: Phase 1 trial of concurrent erlotinib, celecoxib, and reirradiation for recurrent head and neck cancer. Cancer 117:3173–3181, 2011

21. Vlaming MLH, van Esch A, Pala Z, et al: Abcc2 (Mrp2), Abcc3 (Mrp3), and Abcg2 (Bcrp1)

are the main determinants for rapid elimination of methotrexate and its toxic metabolite 7-hydroxymethotrexate in vivo. Mol Cancer Ther 8:3350–3359, 2009

22. Shen B, Dong P, Li D, et al: Expression and function of ABCG2 in head and neck squamous cell carcinoma and cell lines. Exp Ther Med 2:1151–1157, 2011

23. Shi Z, Peng X-X, Kim I-W, et al: Erlotinib (Tarceva, OSI-774) Antagonizes ATP-Binding Cassette Subfamily B Member 1 and ATP-Binding Cassette Subfamily G Member 2–Mediated Drug Resistance. Cancer Res 67:11012–11020, 2007

24. Fox SB, Gasparini G, Harris AL: Angiogenesis: pathological, prognostic, and growth-factor pathways and their link to trial design and anticancer drugs. Lancet Oncol 2:278–289, 2001

25. Pasquier E, Tuset M-P, Street J, et al: Concentration- and schedule-dependent effects of chemotherapy on the angiogenic potential and drug sensitivity of vascular endothelial cells. Angiogenesis 16:373–386, 2013

26. Jain RK: Normalizing tumor microenvironment to treat cancer: bench to bedside to biomarkers. J Clin Oncol 31:2205–2218, 2013

27. Calleri A, Bono A, Bagnardi V, et al: Predictive Potential of Angiogenic Growth Factors and Circulating Endothelial Cells in Breast Cancer Patients Receiving Metronomic Chemotherapy Plus Bevacizumab. Clin Cancer Res 15:7652–7657, 2009

28. Mancuso P, Colleoni M, Calleri A, et al: Circulating endothelial-cell kinetics and viability predict survival in breast cancer patients receiving metronomic chemotherapy. Blood 108:452–459, 2006

29. Munoz R, Shaked Y, Bertolini F, et al: Anti-angiogenic treatment of breast cancer using

metronomic low-dose chemotherapy. Breast 14:466–479, 2005

30. Twardowski PW, Smith-Powell L, Carroll M, et al: Biologic Markers of Angiogenesis: Circulating Endothelial Cells in Patients with Advanced Malignancies Treated on Phase I Protocol with Metronomic Chemotherapy and Celecoxib. Cancer Invest 26:53–59, 2008

31. Bertolini F, Paul S, Mancuso P, et al: Maximum tolerable dose and low-dose metronomic chemotherapy have opposite effects on the mobilization and viability of circulating endothelial progenitor cells. Cancer Res 63:4342–4346, 2003

32. Pasquier E, Kavallaris M, André N: Metronomic chemotherapy: new rationale for new directions. Nat Rev Clin Oncol 7:455–465, 2010

33. Shaked Y, Emmenegger U, Man S, et al: Optimal biologic dose of metronomic chemotherapy regimens is associated with maximum antiangiogenic activity. Blood 106:3058–3061, 2005

34. Colleoni M, Rocca A, Sandri MT, et al: Low-dose oral methotrexate and cyclophosphamide in metastatic breast cancer: antitumor activity and correlation with vascular endothelial growth factor levels. Ann Oncol 13:73–80, 2002

35. Wong NS, Buckman RA, Clemons M, et al: Phase I/II trial of metronomic chemotherapy with daily dalteparin and cyclophosphamide, twice-weekly methotrexate, and daily prednisone as therapy for metastatic breast cancer using vascular endothelial growth factor and soluble vascular endothelial growth factor receptor levels as markers of response. J Clin Oncol 28:723–730, 2010

36. Ogata Y, Sasatomi T, Mori S, et al: Significance of thymidine phosphorylase in metronomic

chemotherapy using CPT-11 and doxifluridine for advanced colorectal carcinoma. Anticancer Res 27:2605–2611, 2007

37. Lee K, Qian DZ, Rey S, et al: Anthracycline chemotherapy inhibits HIF-1 transcriptional activity and tumor-induced mobilization of circulating angiogenic cells. Proc Natl Acad Sci U S A 106:2353–2358, 2009

38. Bocci G, Francia G, Man S, et al: Thrombospondin 1, a mediator of the antiangiogenic effects of low-dose metronomic chemotherapy. Proc Natl Acad Sci U S A 100:12917–12922, 2003

39. Damber J-E, Vallbo C, Albertsson P, et al: The anti-tumour effect of low-dose continuous chemotherapy may partly be mediated by thrombospondin. Cancer Chemother Pharmacol 58:354–360, 2006

40. E. P. Hamilton, G. G. Kimmick, N. Desai, S. Singh, J. O. Hopkins, P. K. Marcom, V. Chadaram, R. Welch, V. N. Trieu, K. L. Blackwell, Duke University Medical Center, Abraxis Bioscience Los, et al: Use of SPARC, EGFR, and VEGFR expression to predict response to nab-paclitaxel (nabP)/carboplatin (C)/bevacizumab (B) chemotherapy in triple-negative metastatic breast cancer (TNMBC). | 2010 ASCO Annual Meeting | Abstracts | Meeting Library. J Clin Oncol 28:(suppl; abstr 1109), 2010

41. Hao Y-B, Yi S-Y, Ruan J, et al: New insights into metronomic chemotherapy-induced immunoregulation. Cancer Lett 354:220–226, 2014

42. Banissi C, Ghiringhelli F, Chen L, et al: Treg depletion with a low-dose metronomic temozolomide regimen in a rat glioma model. Cancer Immunol Immunother 58:1627–1634,

2009

43. Gasparini G: Metronomic scheduling: the future of chemotherapy? Lancet Oncol 2:733–740, 2001

44. Lam T, Hetherington JW, Greenman J, et al: From total empiricism to a rational design of metronomic chemotherapy phase I dosing trials. Anticancer Drugs 17:113–121, 2006

45. Emmenegger U, Kerbel RS: A dynamic de-escalating dosing strategy to determine the optimal biological dose for antiangiogenic drugs. Clin Cancer Res 11:7589–7592, 2005

46. Bocci G, Nicolaou KC, Kerbel RS: Protracted Low-Dose Effects on Human Endothelial Cell Proliferation and Survival in Vitro Reveal a Selective Antiangiogenic Window for Various Chemotherapeutic Drugs. Cancer Res 62:6938–6943, 2002

47. Calleri A, Bono A, Bagnardi V, et al: Predictive Potential of Angiogenic Growth Factors and Circulating Endothelial Cells in Breast Cancer Patients Receiving Metronomic Chemotherapy Plus Bevacizumab. Clin Cancer Res 15:7652–7657, 2009

48. Bertolini F, Shaked Y, Mancuso P, et al: The multifaceted circulating endothelial cell in cancer: towards marker and target identification. Nat Rev Cancer 6:835–845, 2006

49. Beerepoot LV, Mehra N, Vermaat JSP, et al: Increased levels of viable circulating endothelial cells are an indicator of progressive disease in cancer patients. Ann Oncol 15:139–145, 2004

50. Mancuso P, Burlini A, Pruneri G: Resting and activated endothelial cells are increased in the peripheral blood of cancer patients [Internet], 2001Available from: http://bloodjournal.hematologylibrary.org/content/97/11/3658.short

51. Chen C-S, Doloff JC, Waxman DJ: Intermittent metronomic drug schedule is essential for activating antitumor innate immunity and tumor xenograft regression. Neoplasia 16:84–96, 2014

52. Kareva I, Waxman DJ, Lakka Klement G: Metronomic chemotherapy: An attractive alternative to maximum tolerated dose therapy that can activate anti-tumor immunity and minimize therapeutic resistance. Cancer Lett 358:100–106, 2015

53. Bekele BN, Shen Y: A Bayesian approach to jointly modeling toxicity and biomarker expression in a phase I/II dose-finding trial. Biometrics 61:343–354, 2005
